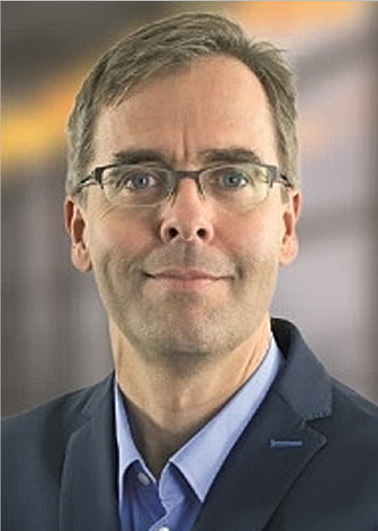# Porphyrin‐Based Metal–Organic Frameworks for Biomedical Applications

**DOI:** 10.1002/anie.201909880

**Published:** 2020-10-12

**Authors:** Jiajie Chen, Yufang Zhu, Stefan Kaskel

**Affiliations:** ^1^ State Key Laboratory of High Performance Ceramics and Superfine Microstructure Shanghai Institute of Ceramics Chinese Academy of Sciences 1295 Dingxi Road Shanghai 200050 China; ^2^ School of Materials Science and Engineering University of Shanghai for Science and Technology 516 Jungong Road Shanghai 200093 China; ^3^ Hubei Key Laboratory of Processing and Application of Catalytic Materials College of Chemical Engineering Huanggang Normal University Huanggang Hubei 438000 China; ^4^ Professur für Anorganische Chemie I Fachrichtung Chemie und Lebensmittelchemie Technische Universität Dresden Bergstrasse 66 Dresden 01062 Germany

**Keywords:** biosensing, metal–organic frameworks, porphyrins, tumor therapy

## Abstract

Porphyrins and porphyrin derivatives have been widely explored for various applications owing to their excellent photophysical and electrochemical properties. However, inherent shortcomings, such as instability and self‐quenching under physiological conditions, limit their biomedical applications. In recent years, metal–organic frameworks (MOFs) have received increasing attention. The construction of porphyrin‐based MOFs by introducing porphyrin molecules into MOFs or using porphyrins as organic linkers to form MOFs can combine the unique features of porphyrins and MOFs as well as overcome the limitations of porphyrins. This Review summarizes important synthesis strategies for porphyrin‐based MOFs including porphyrin@MOFs, porphyrinic MOFs, and composite porphyrinic MOFs, and highlights recent achievements and progress in the development of porphyrin‐based MOFs for biomedical applications in tumor therapy and biosensing. Finally, the challenges and prospects presented by this class of emerging materials for biomedical applications are discussed.

## Graphical abstract

Porphyrin‐based metal–organic frameworks can be designed and constructed for powerful biomedical applications. Recent progress and future expectations are discussed in this Review.

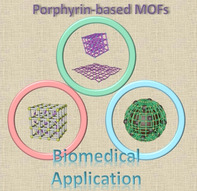

## 1 Introduction

Porphyrins are macromolecular heterocyclic compounds composed of porphin (C_20_H_14_N_4_) substituted by various functional groups at the *meso*‐position or *β*‐position.^[1]^ In addition, free‐base porphyrins can be coordinated with numerous metal ions at the porphyrin center to form metal complexes, also known as metalloporphyrins.^[2]^ In fact, many porphyrins and metalized porphyrin derivatives, such as cytochrome, heme, and chlorophyll, exist in nature and play significant roles in organisms. The family of porphyrins discovered up to now have been comprehensively studied.^[3]^ Due to their large π‐aromatic system, porphyrins display excellent chemical and thermal stability as well as distinct photophysical and electrochemical properties, which can be regulated by the substitution patterns on porphin and the coordinated metal ions. Besides the coordination of metal ions at the porphyrin center, the periphery of porphyrins can also be bound to metal ions.^[4]^ Intricate binding modes enable porphyrins to form desirable molecular cages or framework solids.^[5]^ Porphyrins and porphyrin derivatives are an important class of organic chromophores with considerable absorption in the visible section of the electromagnetic spectrum. Typically, the absorption spectrum shows the strongest absorption around 400–450 nm (Soret band) and a series of absorption bands between 500 and 700 nm (Q‐bands) with gradually reduced intensity.^[1]^

**Figure 1:**
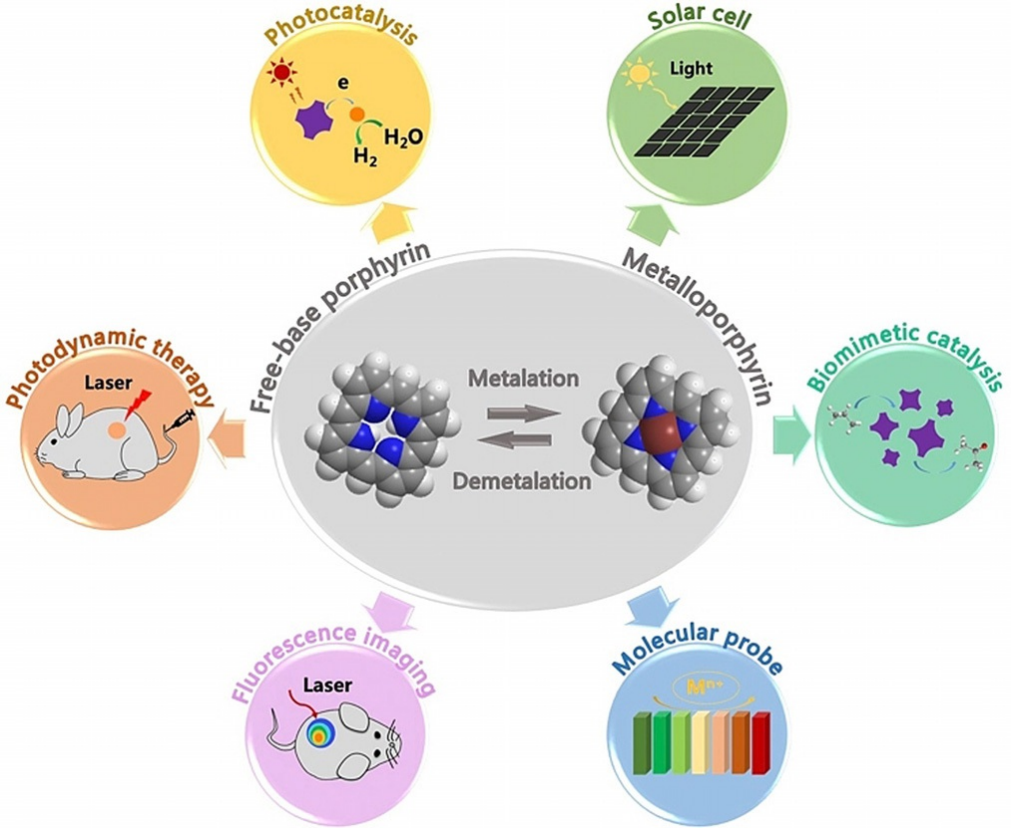
Prominent applications of porphyrins and porphyrin derivatives.

Porphyrins and porphyrin derivatives have been used in various applications due to their characteristics and versatile functions (Figure 1). For instance, owing to their pronounced visible‐light absorption and energy‐transfer properties, porphyrins can be used in light‐harvesting, solar cells, and molecular electronics.^[6]^ The chemical catalytic activity of porphyrins, especially metalloporphyrins, render them efficient photocatalysts, electrocatalysts, and biomimetic catalysts.^[7]^ The regulation of the optical and electronic properties of porphyrins by the coordination of metal ions and the axial coordination of molecules enable their application in molecular recognition and metal‐ion sensing.^[8]^ More importantly, porphyrins are crucial in many biological processes. They have potent biological properties, such as biocompatibility, effective clearance, long residence time in tumors, few side effects, and the mimicking of various biological functions, which are extremely useful for biomedical applications.^[9]^ For example, porphyrins have been widely developed as photosensitizers for photodynamic therapy (PDT). At the same time, their fluorescence characteristics make porphyrin‐based photosensitizers valuable systems for fluorescence imaging‐guided therapy.^[9b]^ However, the drawbacks of most porphyrins and porphyrin derivatives, in particular instability, enzymatic degradation, nonspecific targeting, and propensity to self‐quench under physiological conditions, seriously limit their biomedical applications. To overcome these problems, various carriers have been developed to encapsulate, physically adsorb, or covalently bind porphyrins and porphyrin derivatives, such as micelles,^[10]^ liposomes,^[11]^ carbon nanotubes,^[12]^ inorganic nanoparticles,^[13]^ and polymer nanoparticles.^[14]^

Metal–organic frameworks (MOFs) are a category of hybrid porous coordination polymers with two‐ or three‐dimensional (2D/3D) topologies assembled by the coordination of metal ions/secondary building units (SBUs) with organic linkers. As an emerging class of material, MOFs have recently attracted increasing attention because of their multiple merits including ultrahigh porosity,^[15]^ tunable pores, adjustable structure and composition, unsaturated metal sites, and functional diversity.^[16]^ Furthermore, the particle size of MOFs can be easily decreased down to the nanoscale, and nanoscale MOFs (NMOFs) are suitable as delivery systems. Compared with other types of carriers, NMOFs are more promising as versatile nanoplatforms for biomedical applications, such as drug delivery, tumor therapy, bioimaging, and biosensing.^[17]^

Accordingly, it was proposed that the integration of porphyrins into MOFs could lead to multifunctional carriers having promising characteristics and versatile functional components. From this perspective, porphyrin‐based MOFs are ideal biomaterials and their full potential has not yet been tapped. Moreover, the conceptual construction of porphyrin‐based MOFs matches well with advanced design concepts for highly active biomaterials that maximize integrated functional building units while minimizing inactive constituents.^[18]^ Therefore, much effort has been devoted to the development of various porphyrin‐based MOF platforms for biomedical applications. The loading of porphyrins into MOF channels and the decoration of porphyrins on the MOF surface are efficient strategies for the synthesis of porphyrin@MOFs that enhance the stability of the porphyrin and facilitate potential applications.^[19]^ Interestingly, free‐base porphyrins and metalloporphyrins can also be used as organic linkers to assemble porphyrinic and metalloporphyrinic MOFs, respectively. In this way, the self‐aggregation and self‐quenching of the porphyrins can be prevented and the physicochemical properties improved. Due to the highly active frameworks and improved performance of porphyrins in MOFs, porphyrin‐based MOFs present a promising opportunity for biomedical applications.

Numerous excellent reviews in the past two decades have separately addressed porphyrins and MOFs, and their respective potential biomedical applications. However, so far a comprehensive review linking porphyrins to MOFs for biomedical applications has been missing. In this Review, we summarize the latest achievements and progress in the field of porphyrin‐based MOFs, from synthesis strategies all the way to functions and applications. We discuss numerous biomedical applications to provide a concise overview and motivation for further work in this promising direction. Furthermore, we also discuss the challenges and prospects for biomedical applications of porphyrin‐based MOFs.

## 2 Synthesis of Porphyrin‐Based MOFs

A tremendous number of MOFs, for example ZIFs (zeolite imidazolate frameworks),^[20]^ MILs (Matériaux de l′Institut Lavoisier),^[21]^ and UiOs (Universitetet i Oslo),^[22]^ have been successfully synthesized, and provide efficient platforms to encapsulate or deliver pharmaceuticals, imaging agents, and enzymes. For developing biomedical nanoplatforms, the downsizing of MOFs to the nanoscale (10–100 nm) is essential because of the significant influence on the size‐dependent function and biodistribution of administered particles. To date, a variety of strategies have been proposed for the synthesis of NMOFs and have been summarized in several reviews.^[23]^ Particularly for the synthesis of NMOFs, several efficient methods have also been developed, including hydrothermal/solvothermal, sonochemical, and mechanochemical methods, microwave‐assisted synthesis, and reverse microemulsion. However, there have been no review articles on the synthesis of porphyrin‐based MOFs.

Typically, porphyrin‐based MOFs can be categorized as porphyrin@MOFs and porphyrinic MOFs, according to the distinct structures assembled from porphyrins and MOFs. For porphyrin@MOFs, free‐base porphyrins or metalloporphyrins are integrated as guest molecules into MOFs via encapsulation in the pores or through adsorption or grafting on the surface. In porphyrinic/metalloporphyrinic MOFs, free‐base porphyrins/metalloporphyrins act as organic linkers and coordinate with metal ions or SBUs. In addition, porphyrinic/metalloporphyrinic MOFs can be combined with other functional components, like magnetic nanoparticles, photothermal agents, and fluorescent quantum dots, to form multifunctional platforms. The morphology, structure, and composition of porphyrin‐based MOFs are vital for biomedical applications, and much effort has been devoted to synthesizing porphyrin‐based MOFs to meet the requirements of biomedical applications.

### 2.1 Synthesis of Porphyrin@MOFs

**Figure 2:**
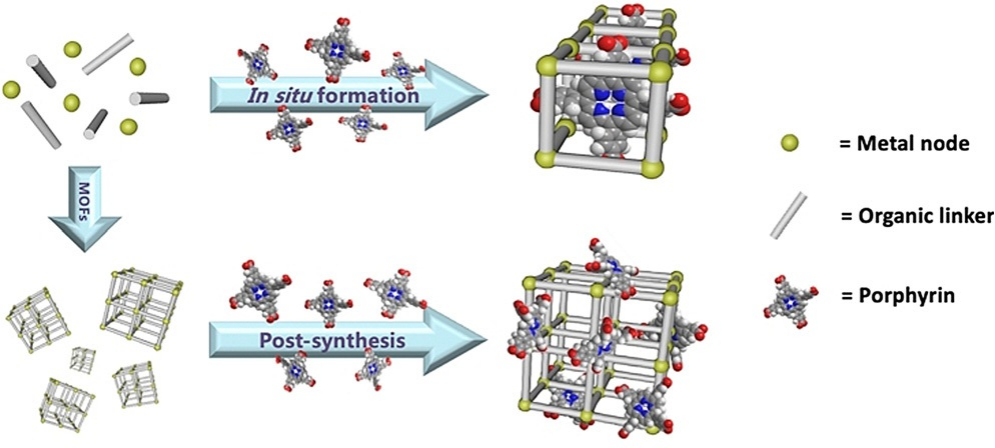
Two different processes for integrating porphyrins in MOFs: in situ formation and post‐synthesis.

To synthesize porphyrin@MOFs, free‐base porphyrins or metalloporphyrins are typically encapsulated into the pores or decorated on the surface of MOFs, and in situ formation and post‐synthesis methods are mainly used for these porphyrin‐integrating processes (Figure 2).^[19]^ The in situ formation method refers to a one‐pot reaction of MOF precursors (i.e., metal nodes and organic linkers) with free‐base porphyrins/metalloporphyrins. During the formation of the frameworks, free‐base porphyrins/metalloporphyrins are entrapped inside MOFs like a “ship in a bottle”. In contrast, post‐synthesis methods realize the encapsulation into pores or grafting on the surface of MOFs through host–guest reactions between synthetic MOFs and free‐base porphyrins/metalloporphyrins; these interactions include hydrogen bonding, van der Waals forces, electrostatic interactions, π–π stacking interactions, covalent bonds, and even coordinative bonds.^[24]^ To date, a variety of porphyrin@MOFs have been successfully synthesized, and some typical examples are summarized in Table 1.

**Table 1:** Typical examples of porphyrin@MOFs for biomedical applications.

MOF	Porphyrin	Method	Biomedical application	Ref.
UiO‐66	DTPP(Zn)‐I_2_	in situ formation	PDT	[26a]
UiO‐66	TCPP	in situ formation	PDT	[26b, 111]
HKUST‐1	TMPyP	in situ formation	PDT; fluorescence imaging	[45]
HKUST‐1	TCPP(Fe)	in situ formation	DNA sensing	[86a]
HKUST‐1	hemin	in situ formation	detection of H_2_O_2_, glucose	[112]
Fe‐MIL‐88	hemin	in situ formation	detection of TB	[26c]
Fe‐MIL‐88	hemin	in situ formation	detection of the FGFR3 gene mutation	[26d]
Fe‐MIL‐88	hemin	in situ formation	immunoassay of PSA	[89]
Tb‐MOF	hemin	in situ formation	colorimetric immunoassay of AFP	[113]
TTA‐UC MOF	TCPP(Pd)	in situ formation	ultralow‐power in vivo imaging	[77]
*rho*‐ZMOF	TMPyP(Pt)	in situ formation	anion‐selective sensing	[8c]
UiO‐66	TPP‐SH	post‐synthesis	PDT	[111]
UiO‐AM	TAPP	post‐synthesis	PDT	[114]
ICR‐2	TPPPi(R), R=methyl, isopropyl, phenyl	post‐synthesis	PDT	[27]
MIL‐101(Al)‐NH_2_	hemin	post‐synthesis	detection of H_2_O_2_, glucose	[93]
Cu‐MOF‐74	hemin	post‐synthesis	2,4,6‐trichlorophenol sensing	[115]

[a] TB=thrombin; PSA=prostatic specific antigen; AFP=alpha‐fetoprotein.

When in situ formation methods are used to encapsulate porphyrins in the interior of MOFs, small pore windows can prevent the leakage of porphyrins as well as facilitate the diffusion of smaller molecules. Eddaoudi et al. were the first to successfully decorate MOFs with porphyrins by in situ formation in 2008.^[25]^ The encapsulation of cationic 5,10,15,20‐tetrakis(1‐methyl‐4‐pyridinio)porphyrin (TMPyP) in *rho*‐ZMOF (indium‐imidazole‐dicarboxylate‐based zeolite‐like metal‐organic framework) was achieved via the one‐pot reaction of In^3+^, 4,5‐imidazoledicarboxylic acid (H_3_ImDC), and TMPyP in an *N*,*N*′‐dimethylformamide (DMF)/acetonitrile (CH_3_CN) solution. Furthermore, Mn, Co, Cu, and Zn ions could be used for the metalation of the encapsulated free‐base porphyrins.

Due to the simplified synthesis and high efficiency of porphyrin encapsulation by the in situ formation method, many groups have attempted to synthesize porphyrin@MOFs as functional platforms for biomedical applications. Among them, porphyrin@UiO‐66 and porphyrin@MIL‐88(Fe) have been successfully synthesized, in which the free‐base porphyrins/metalloporphyrins are protected by encapsulation and which also contain biocompatible Zr and Fe ions.^[26]^ For example, Dong et al. reported the synthesis of a porphyrin@UiO‐66 material in which UiO‐66 is used to encapsulate 5,15‐di(4‐carboxyphenyl)‐10,20‐bis(4‐iodophenyl)porphyrin zinc(II) (DTPP(Zn)‐I_2_) by the in situ formation method.^[26a]^ The loading of DTPP(Zn)‐I_2_ in UiO‐66 was estimated to be ca. 1.4 wt %, which was much higher than that obtained by the normal impregnation method (0.06 wt %).

For post‐synthesis functionalization, the construction of porphyrin@MOFs generally requires appropriate interactions between the porphyrins and MOFs, and the following issues require consideration:

1) MOFs should be activated to eliminate solvent molecules in the pores or channels before decoration.

2) The size and shape of the porphyrins should match the dimensions of the MOFs’ pores or channels; this allows the entrance of porphyrins throughout the integration process.

3) Bond formation should be triggered between the porphyrin and the framework.

Demel et al. developed a phosphinate‐based MOF (ICR‐2, Inorganic Chemistry Rez No. 2), which could be decorated with anionic 5,10,15,20‐tetrakis(4‐R‐phosphinatophenyl)porphyrins (TPPPi(R), R=methyl, isopropyl, phenyl) by interaction with the unsaturated metal sites on the ICR‐2 surface.^[27]^ The fraction of TPPPi(R) decorated on the MOFs was higher than that possible with the commercially available 5,10,15,20‐tetrakis(4‐carboxyphenyl)porphyrin (TCPP). This might be explained by the fact that the bond between the metal ions and the phosphinic groups in TPPPi(R) is stronger than that between the metal ions and the carboxylic groups in TCPP.

### 2.2 Synthesis of Porphyrinic MOFs

Porphyrins are macromolecular heterocyclic compounds that can be used as organic linkers to coordinate with metal ions or SBUs to form porphyrinic MOFs. Such porphyrinic MOFs feature porphyrin functionality but also provide high porosity to host further secondary functional components. In 1991 Robson et al. described the first porphyrinic MOFs,^[28]^ which were assembled from 5,10,15,20‐tetrakis(4‐pyridyl)porphyrin palladium(II) (TPyP(Pd)) as linkers and Cd^2+^ ions as nodes. Interestingly, SBUs, also termed metal clusters, could be formed through stable metal–oxygen bonds or metal–nitrogen bonds. Such units play a key role in improving structural stability, generating more coordination sites, promoting framework extension, preventing structural interpenetration, and expanding pores in MOFs. Therefore, SBUs as nodes can be used to construct robust porphyrinic MOFs with high stability, and recently increasing attention has arisen due to their potential biomedical applications.^[29]^ In 2002, Suslick et al. reported the first stable porphyrinic MOF (PIZA‐1, porphyrinic Illinois zeolite analogue No. 1) with SBUs as nodes,^[30]^ which was synthesized via self‐assembly of trinuclear Co^II^‐carboxylate clusters and Co^III^‐metalloporphyrin linkers through a solvothermal treatment. These porphyrinic MOFs have large and refillable tridirectional channels and exhibit significant hydrophilic character.

The rational choice of porphyrins plays a vital role in regulating the pores, shapes, and sizes of porphyrinic MOFs during the synthesis.^[31]^ Carboxy‐based porphyrins, such as TCPP and its metalized molecules (TCPP(M)), have been widely used as linkers to synthesize porphyrinic MOFs.^[31a]^ Generally, free‐base porphyrins can form porphyrinic MOFs without metal chelation, while the cores of free‐base porphyrins can be pre‐metalized or metalized by in situ chelation or post‐synthesis chelation with various metal ions to generate metalloporphyrinic MOFs. On the other hand, it is interesting that weakly coordinated metal ions can be replaced by other more strongly binding metal ions,^[32]^ and thereby there are alternative approaches for the synthesis of stable porphyrinic MOFs.

**Figure 3:**
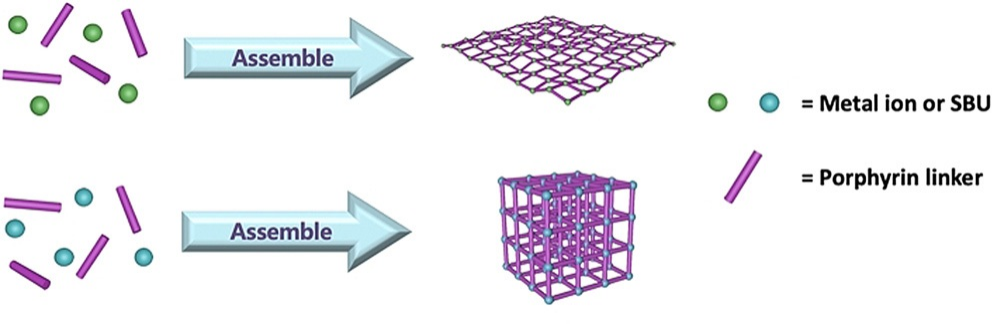
Synthesis of porphyrinic MOFs with 2D or 3D structure.

To date, various metal nodes, such as Cu, Zn, Co, and Cd ions, have been used to assemble with TCPP or TCPP(M) linkers to form 2D MOFs (Figure 3).[^6e^, ^33^] For example, Zhang et al. reported the first surfactant‐assisted method to synthesize homogeneous ultrathin 2D MOF nanosheets (Zn‐TCPP) with a thickness of less than 10 nm.^[33c]^ In 2D Zn‐TCPP nanosheets, one TCPP ligand is linked to four Zn paddlewheel metal nodes (Zn_2_(COO)_4_) and the TCPP ligand is metalized by Zn ions in the course of the formation. This strategy was also used to prepare several different ultrathin 2D porphyrinic MOF nanosheets, including Cu‐TCPP, Cd‐TCPP, and Co‐TCPP. Recently, Hong et al. utilized TCPP(Fe) ligands to link various divalent metal ions (Zn, Co, and Cu) to construct a series of 2D metalloporphyrinic MOF nanosheets.^[33e]^

The coordination of TCPP or TCPP(M) with Mn‐, Fe‐, Zr‐, and Hf‐based clusters results in the formation of porphyrinic MOFs with typical 3D topologies (Figure 3 **)**.^[34]^ Zhou et al. synthesized several porphyrinic MOFs (porous coordination networks, PCNs) with different topologies by a solvothermal method;^[35]^ porphyrinic MOFs such as PCN‐222, PCN‐223, PCN‐224, and PCN‐225 were built from TCPP or TCPP(M) (M=Ni, Cu, Zn, Co, Mn, or Fe) and Zr_6_ clusters. Importantly, the Zr_6_ clusters with high connectivity and high charge density form very strong Zr−O bonds, which contributes to the high stability of PCNs in acidic or alkaline aqueous solutions. By analogy with PCNs, Yaghi et al. synthesized MOF‐525 and MOF‐545 by coordinating TCPP with Zr_6_O_4_(OH)_4_ and Zr_6_O_8_(H_2_O)_8_ units, respectively.^[34d]^ Further, MOF‐525 and MOF‐545 could be post‐synthetically metalized with Fe^3+^ and Cu^2+^ to yield metalloporphyrinic MOFs without losing their high surface area and chemical stability.

Interestingly, many studies have proposed the coordination between metal nodes and two or more kinds of linkers to construct multivariate MOFs for introducing multiple functionalities into MOFs.^[36]^ Here, the introduction of other organic linkers into porphyrinic MOFs can regulate the physicochemical properties of porphyrins or endow frameworks with additional functions. For instance, Zhou et al. developed a photochromic MOF (SO‐PCN, singlet oxygen‐generating porous coordination network), which was constructed by self‐assembly of dual linkers, 1,2‐bis(2‐methyl‐5‐(pyridin‐4‐yl)thiophen‐3‐yl)cyclopent‐1‐ene (BPDTE) and TCPP, with Zn nodes.^[36a]^ Due to the integration of the photochromic switch BPDTE into the porphyrinic MOF, SO‐PCN displayed reversible control over the photosensitization of porphyrins by an energy transfer process upon irradiation at specific wavelengths, and thereby met the requirements for light‐controlled applications.

Various porphyrinic MOFs with different structures have been synthesized due to the diversity of porphyrins and metal nodes. Similar to other MOFs, the downsizing of porphyrinic MOF crystals to the nanoscale can also be achieved to improve physiochemical and biological properties for biomedical applications. Solvothermal synthesis is by far the most common method for synthesizing porphyrinic NMOFs.[^35d^, ^37^] Here, the types of metal nodes and porphyrins, the reaction conditions, solvent species, stoichiometry, molecular modulators, temperature, and reaction time are the key factors to control the size and morphology of porphyrinic NMOFs, which should be carefully considered in synthesis process. Table 2 summarizes typical conditions for the synthesis of porphyrinic MOFs for biomedical applications.

**Table 2:** Typical examples of porphyrinic MOFs for biomedical applications.

MOF	Metal ion	Porphyrin	Other components	Biomedical application	Ref.
PCN‐221	Zr^4+^	TCPP	MTX	chemotherapy	[64]
PCN‐222	Zr^4+^	TCPP	ss‐DNA‐tagged antibody (Ab‐DNA)	PSA sensing	[88]
PCN‐222	Zr^4+^	TCPP		label‐free phosphoprotein (α‐casein) sensing	[91]
PCN‐222	Zr^4+^	TCPP		reversible colorimetric and fluorescent low‐pH response	[116]
PCN‐222(Fe)	Zr^4+^	TCPP(Fe)	SA	DNA sensing	[86b]
PCN‐222(Pd)	Zr^4+^	TCPP(Pd)		fluorescence “turn‐on” sensing of Cu^2+^	[99]
PCN‐222/PCN‐224	Zr^4+^	TCPP		PDT	[117]
PCN‐224	Zr^4+^	TCPP	DNA aptamer (AS1411), therapeutic DNA (CpG)	immunotherapy; targeted imaging	[52a]
PCN‐224	Zr^4+^	TCPP	FA	targeted PDT	[35d]
PCN‐224	Zr^4+^	TCPP		NIR light induced attenuation of aggregation and neurotoxicity of Aβ	[105]
PCN‐225	Zr^4+^	TCPP		fluorescent pH sensing	[35b]
MOF‐525	Zr^4+^	TCPP		nitrite detection	[118]
MOF‐525	Zr^4+^	TCPP		luminescent sensing of Cu^2+^	[97]
MOF‐525(Zn)	Zr^4+^	TCPP(Zn)		PKA activity assay	[92]
NPMOF	Zr^4+^	TCPP	DOX	chemotherapy, PDT; fluorescence imaging	[37a]
NT	Zr^4+^	TCPP	TBAPy ligand, FA	PDT	[36e]
Zr(TBAPy)_5_(TCPP)	Zr^4+^	TCPP	TBAPy ligand	detection of H_2_S, S^2‐^; fingerprint identification	[36c]
PorMOF	Zr^4+^	TCPP(Fe)	SA	detection of telomerase	[90]
TBP‐NMOF	Zr^4+^	TBP/TBP(Cu)		PDT, immunotherapy	[70]
Zr‐FeP	Zr^4+^	TBP(Fe)	heat shock protein 70 siRNA	PDT, low‐temperature PTT; PTI, PAI, CT	[66]
DBP‐UiO	Hf^4+^	DBP		PDT	[42]
R‐UiO	Hf^4+^	DBP(Pt)	QPDC‐NH_2_ ligand, RBITC	intracellular O_2_ sensing	[36b]
DBC‐UiO	Hf^4+^	DBC		PDT	[47]
Hf‐TCPP	Hf^4+^	TCPP	C_18_PMH‐PEG	PDT, RT	[44]
TCPC‐UiO	Hf^4+^	TCPC	BDC ligand	PTT, PDT; CT, PTI, PAI	[36f]
TBC‐Hf/TBP‐Hf	Hf^4+^	TBC/TBP	IDOi (INCB24 360)	PDT, immunotherapy	[69a]
MOF‐2	Al^3+^	TCPP(Cu)		PDT	[58]
PAC	Al^3+^	TCPP(Cu)		detection of intracellular H_2_S	[94]
Gd‐TCPP	Gd^3+^	TCPP		PDT; MRI	[33d]
Mn‐TCPP	Mn^3+^	TCPP		controllable PDT; MRI, fluorescence imaging	[59b]
Fe‐TBP	Fe^3+^	TBP		PDT, immunotherapy	[59a]
Cu‐TCPP	Cu^2+^	TCPP		PTT, PDT; MRI, PTI	[67]
Cu‐TCPP	Cu^2+^	TCPP		selective generation of ^1^O_2_	[60]
Cu‐TCPP	Cu^2+^	TCPP		fluorometric determination of the target DNAs	[87]
Cu‐TCPP(Fe)	Cu^2+^	TCPP(Fe)	cisplatin	chemotherapy, highly increased generation of ROS	[56b]
NP‐1	Cu^2+^	TCPP(Zn)		controllable PDT	[59a]
SO‐PCN	Zn^2+^	TCPP	photochromic switch molecule (BPDTE)	reversible control of ^1^O_2_ generation	[36a]
Pd‐MOF	Pd^2+^	TPyP	hydrogen gas	hydrogenothermal therapy; PAI	[74]

### 2.3 Synthesis of Composite Porphyrinic MOFs

To endow porphyrinic MOFs with multifunctionality, the incorporation of functional components into porphyrinic MOFs to construct composite porphyrinic MOFs is a useful strategy. To date, various functional components (e.g., magnetic nanoparticles, upconversion nanoparticles (UCNPs), biomolecules) have been introduced to form composite porphyrinic MOFs for biomedical applications.^[38]^ For example, Zhang et al. reported the growth of porphyrinic MOFs on the surfaces of polydopamine nanoparticles, graphene oxide sheets, and gold nanorods to form core–shell composite porphyrinic MOFs.^[38g]^ This strategy was driven by controlling the coordination interactions between the functional groups of the nanostructures and the Zr metal nodes, which not only prevented the self‐nucleation of MOFs in solution but also controlled the thickness of porphyrinic MOFs. More interestingly, Li et al. reported in situ growth of porphyrinic MOFs on polyvinylpyrrolidone (PVP)‐coated lanthanide‐doped UCNPs to form UCNPs/PMOF heterodimers with asymmetric compositions.^[38c]^

In this context, the surface interactions including electrostatic forces, hydrophobic interactions, and covalent or coordinate bonds, are critical factors to control composite porphyrinic MOFs with well‐defined morphology. Therefore, surface modifications are necessary for the fabrication of composite porphyrinic MOFs. The intricate interactions in composite porphyrinic MOFs also affect the degradation characteristics. For example, the high affinity of Zr cations to phosphate anions can result in the disassembly of Zr‐based porphyrinic MOFs. Ren et al. evaluated the degradation degree of platinum‐decorated PCN‐224 (PCN‐224‐Pt) and PCN‐224 in phosphate‐buffered saline (PBS).^[39]^ The results indicated that PCN‐224‐Pt degraded and released TCPP ligands more slowly than PCN‐224 due to the strong covalent interaction between Pt and PCN‐224. Moreover, decorating the surface of MOFs with biocompatible materials (e.g., polyethylene glycol (PEG), PVP) can improve their stability and prolong circulation time in biological environments. Although a variety of composite porphyrinic MOFs have been synthesized, the complicated synthesis process, low yield, and highly wasteful production still restrict their development in biomedical applications. Table 3 summarizes some typical examples of composite porphyrinic MOFs for biomedical applications.

**Table 3:** Typical examples of composite porphyrinic MOFs for biomedical applications.

Composite	Porphyrinic MOF	Functional components	Biomedical application	Ref.
PCN‐224‐Pt	PCN‐224	Pt nanoparticles	PDT	[39]
mCGP	PCN‐224	GOx, catalase, tumor cell membrane	cancer targeted starvation, PDT	[52b]
l‐Arg@PCN@Mem	PCN‐224	l‐arginine, tumor cell membrane	gas therapy, PDT	[52c]
Dox/UCMOFs	PCN‐224	UCNPs	chemotherapy, PDT	[38c]
MOF‐UCNP	PCN‐224	UCNPs	PDT	[38d]
UiO‐66(OH)_2_@PCN	PCN‐224	UiO‐66(OH)_2_	Cu^2+^ sensing	[98]
RB‐PCN	PCN‐224	Fe_3_O_4_, RBITC	broad‐range pH sensor for fluorescence imaging	[102]
AuNR@MOFs@CPT	Zr‐TCPP	Au nanorods, CPT	PDT, PTT, chemotherapy; fluorescence imaging	[38a]
PDA@MOF	Zr‐TCPP	PDA nanoparticles	PTT, PDT	[38g]
Fe_3_O_4_@C@PMOF	Zr‐TCPP	Fe_3_O_4_@C nanospheres	PTT, PDT; MRI, fluorescence imaging	[79]
NMOF‐SNO	Zr‐TCPP(Mn)	S‐nitrosothiol	gas therapy, PTT; MRI	[81]
MOF‐525‐PEDOT NTs	MOF‐525	poly(3,4‐ethylenedioxythiophene) nanotubes	detection of dopamine	[95]
MOF‐525/MPC	MOF‐525	macroporous carbon	detection of luteolin	[119]
Cu‐TCPP(Co)/MWCNTs	Cu‐TCPP(Co)	multi‐walled carbon nanotubes	detection of nitrite and H_2_O_2_	[38f]
Cu‐TCPP(Fe)/GOx	Cu‐TCPP(Fe)	GOx	antibacterial activity and in vivo wound healing	[104]
Au NPs/Cu‐TCPP(M)	Cu‐TCPP(M), M=Fe, Co	Au nanoparticles	detection of glucose	[38b]
Ag/Cu‐TCPP(Cu)	Cu‐TCPP(Cu)	Ag nanoparticles	antibacterial activity and in vivo wound healing	[103]
MOFs/CS‐rGO	Cu‐hemin	chitosan‐functionalized reduced graphene oxide	detection of H_2_O_2_	[120]
Cu‐H MOFs/NECF	Cu‐hemin	nitrogen‐containing melamine carbon foam	detection of trichlorfon	[96]
CHC‐PZM@HA	Zr‐TCPP	α‐cyano‐4‐hydroxycinnamate, HA	PDT	[51]
L/AuNP/(Fe‐P)n‐MOF	Fe‐TCPP	Au nanoparticles, DNA	T4 PNK activity detection	[121]

## 3 Biomedical Applications of Porphyrin‐Based MOFs

**Figure 4:**
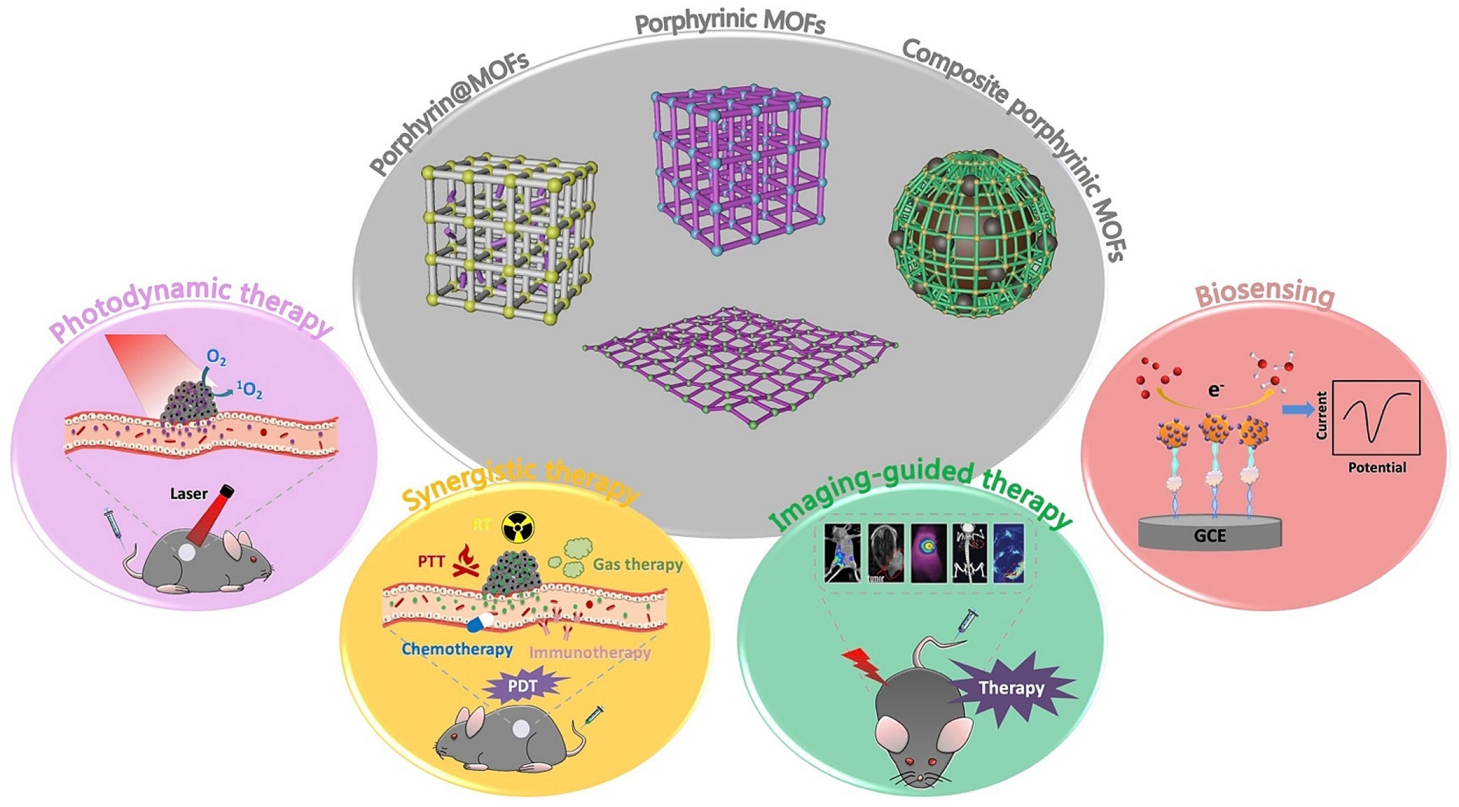
The main biomedical applications of porphyrin‐based MOFs. Reproduced with permission.^[79]^ Copyright 2017, Nature Publishing Group. Reproduced with permission.^[66]^ Copyright 2018, Wiley‐VCH.

Porphyrin‐based MOFs offer a variety of attractive features making use of the integration of porphyrins into MOFs, in particular excellent photophysical and electrochemical properties, porous structure, modular functionalization, and biocompatibility. These characteristics are beneficial for applications of porphyrin‐based MOFs in biomedicine, which have attracted more and more attention. Nowadays, numerous interesting biomedical applications, such as drug delivery, tumor therapy, bioimaging and biosensing, have been developed for porphyrin‐based MOFs. In the following, we summarize recent progress in the area of biomedical applications of porphyrin‐based MOFs, including photodynamic therapy (PDT), synergistic therapy, imaging‐guided therapy, and biosensing (Figure 4).

### 3.1 Photodynamic Therapy with Porphyrin‐Based MOFs

PDT is an efficient tumor therapy strategy that requires three essential factors: photosensitizer, light, and molecular oxygen in cells.^[40]^ As a result, PDT generates highly cytotoxic reactive oxygen species (ROS) under light irradiation. ROS including singlet oxygen (^1^O_2_), superoxide anion radical (O_2_^−.^), and hydroxyl radical (^.^OH) can induce tumor cell death through apoptosis and/or necrosis, and even tumor immunity. By intravenous injection of photosensitizers for accumulation in tumor tissue and local exposure of tumor sites to light, PDT can selectively destroy tumor cells while damage to the surrounding normal cells and tissues is minimized.^[41]^ This approach profits from being non‐invasive and has fewer side effects (e.g., radiation damage, drug toxicity) than conventional surgery, radiotherapy, and chemotherapy.

Porphyrin and its derivatives are excellent photosensitizers for PDT and several have been approved for clinical trials. For porphyrin‐based MOFs, the high porphyrin loading capacity and free diffusion of oxygen and ROS in the porous material allow for highly efficient PDT. Furthermore, the biocompatibility and biodegradability of MOFs result in enhanced biosafety during PDT. Since the first report on porphyrinic NMOFs for potential tumor PDT by Lin et al. in 2014,^[42]^ numerous porphyrin‐based MOFs have been designed for PDT. The most important advances are summarized in Tables 1– 3.

#### 3.1.1 Enhanced Photodynamic Therapy

In general, the key factor for PDT is the quantity of ^1^O_2_ generated by photosensitizers upon light irradiation, which can be regulated by the efficiency of the intersystem crossing (ISC) of the photosensitizers from the singlet state (S_1_) to the triplet state (T_1_).^[43]^ Interestingly, the introduction of heavy atoms into photosensitizers is known to enhance ISC and is the “heavy atom effect”. In porphyrin‐based MOFs, the interactions between porphyrins and metal ions can improve the photodynamic properties of porphyrins on account of the “heavy atom effect”, and thereby increase the production of ^1^O_2_.^[40a]^

**Figure 5:**
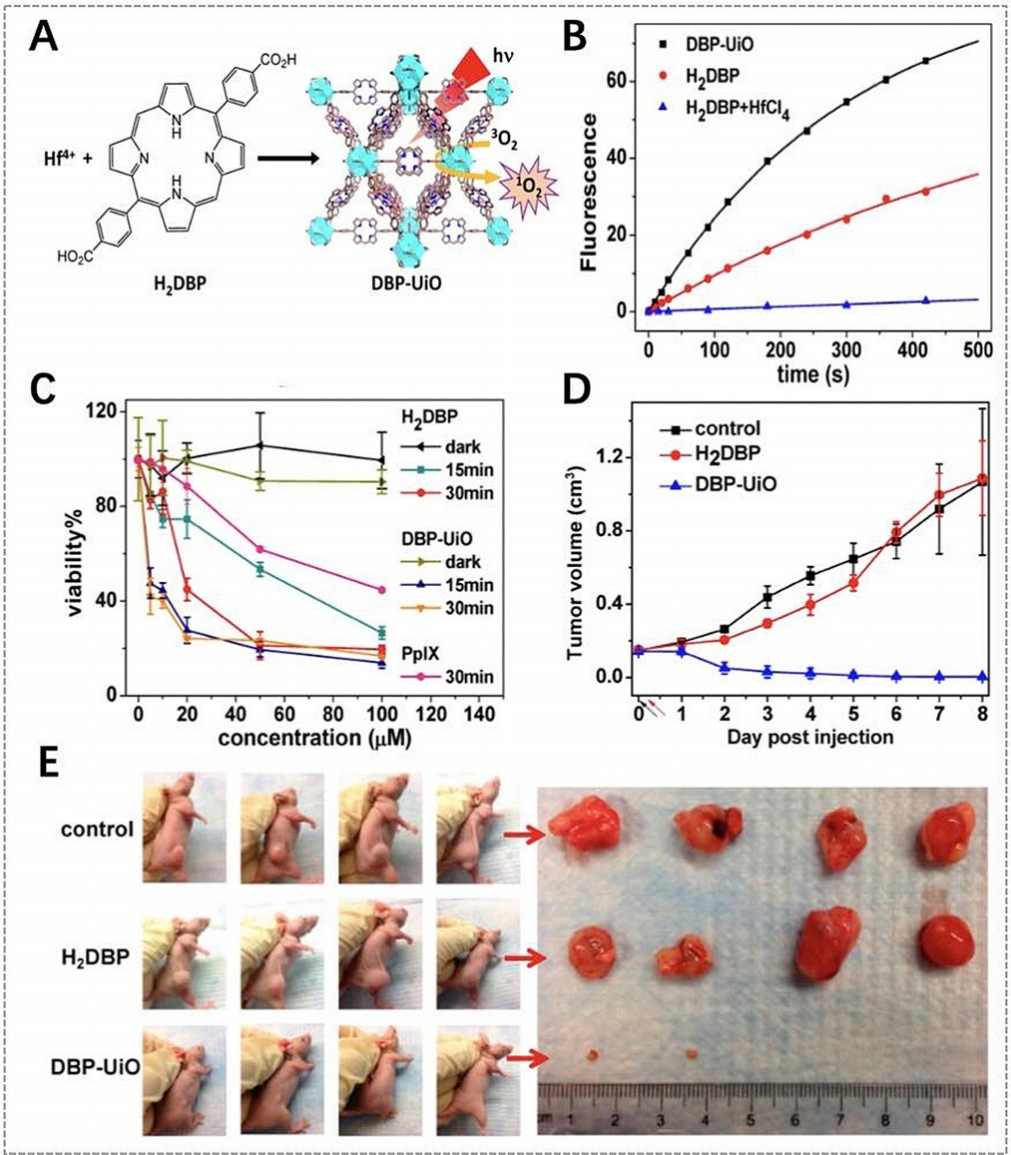
A) Synthesis of DBP‐UiO NMOFs and generation of ^1^O_2_. B) ^1^O_2_ generation of DBP‐UiO, H_2_DBP, and H_2_DBP+HfCl_4_ detected by Singlet Oxygen Sensor Green (SOSG) assay. C) In vitro PDT cytotoxicity of different components (PpIX=protoporphyrin IX). D) In vivo tumor volume changes after PDT in the presence of different components (black and red arrows refer to the injection and irradiation time points, respectively). E) Photographs of the mice and the corresponding tumors after PDT. (A–E) Reproduced with permission.^[42]^ Copyright 2016, American Chemical Society.

The first report on porphyrinic MOFs for PDT was the use of DBP‐UiO nanoplatelets, which were assembled from 5,15‐di(*p*‐benzoato)porphyrin (DBP) as linkers and Hf^4+^ ions as metal nodes (Figure 5).^[42]^ DBP‐UiO nanoplates had a high porphyrin loading capacity of 77 wt %, and showed at least twice as efficient ^1^O_2_ production as free DBP. Correspondingly, DBP‐UiO nanoplates presented highly enhanced PDT efficacy against human head and neck tumor cells (SQ20B) compared to free DBP. Therefore, the coordination of porphyrin linkers with heavy metal nodes in porphyrinic MOFs lead to an enhancement on PDT, caused by the “heavy atom effect”, and the same phenomenon was observed in other porphyrinic MOFs.[^33d^, ^39^, ^44^] For example, Gd‐TCPP MOF nanosheets, which were linked by TCPP with Gd ions, showed improved photosensitive activity compared to free TCPP.^[33d]^ On the other hand, the encapsulation of porphyrins in MOFs can also enhance ISC together with the ^1^O_2_ generation. Lei et al. encapsulated TMPyP into HKUST‐1 and found that the narrowed S_1_–T_1_ energy gap of TMPyP caused by the interaction between the encapsulated TMPyP and Cu^2+^ nodes enhanced the ISC. As a result, TMPyP@HKUST‐1 exhibited higher ^1^O_2_ production capability compared to free TMPyP, and thereby induced higher phototoxicity to tumor cells.^[45]^

To optimize the photophysical properties and PDT efficacy of porphyrin‐based MOFs, the reduction of porphyrins to chlorins by the hydrogenation of the cross‐conjugated double bond of porphyrin ring is also an effective strategy, which could result in a red‐shift of absorption and an increase of the extinction coefficient.^[46]^ Lin et al. described the first chlorin‐based NMOFs (DBC‐UiO), which were synthesized from Hf^4+^ nodes and 5,15‐di(*p*‐benzoato)chlorin (DBC), and found an enhanced PDT effect for colon tumors.^[47]^ There was a 13 nm red shift in absorption and an 11‐fold augmentation in the extinction coefficient of the longest‐wavelength Q band. Consequently, DBC‐UiO NMOFs were three times as efficient as DBP‐UiO NMOFs in the generation of ^1^O_2_ and exhibited a much stronger PDT effect in dual colon tumor cell lines (CT26 and HT29). Therefore, the use of chlorins to construct NMOFs provides nanoplatforms for realizing highly efficient PDT owing to the improved photophysical properties of the reduced porphyrins.

Generally, the accumulation of photosensitizers in tumor tissues and further uptake by tumor cells facilitates efficient PDT. It is well known that the size of NMOFs is of crucial importance during cellular uptake.^[48]^ In many studies the MOF particle size has been optimized to further enhance PDT efficacy. For example, Zhou et al. synthesized PCN‐224 with sizes ranging from 30 to 190 nm to investigate the effect of particle size on cellular uptake and PDT efficacy.^[35d]^ These PCN‐224 nanoparticles showed different uptake levels in Hela cells, and the 90 nm sized PCN‐224 had a maximal cellular uptake amount, as determined by inductively coupled plasma mass spectrometry (ICP‐MS). Furthermore, the PDT for Hela cells indicated that the 90 nm‐sized PCN‐224 induced 81 % cell apoptosis after PDT treatment, which was much higher than that induced by other sized PCN‐224, suggesting that the size‐dependent cellular uptake of NMOFs determines PDT efficacy.

#### 3.1.2 Light‐Controlled Photodynamic Therapy

Controllable generation of ^1^O_2_ is useful for enhancing PDT efficacy, which requires the release of cytotoxic ^1^O_2_ only in tumor sites and less damage to normal cells and tissues.^[49]^ Light, as an essential exogenous condition for PDT, has been utilized to control the generation of ^1^O_2_ from porphyrin‐based MOFs in PDT, and thereby enhance PDT efficacy and reduce toxic side effects. For example, Zhou et al. synthesized photochromic SO‐PCN MOFs by the integration of the photochromic switch molecule BPDTE (1,2‐bis(2‐methyl‐5‐(pyridin‐4‐yl)thiophen‐3‐yl)cyclopent‐1‐ene) into porphyrinic MOFs. The SO‐PCN MOFs showed a reversible control of ^1^O_2_ production via a competitive energy transfer pathway upon specific wavelength irradiation.^[36a]^ Here, the open and closed state of BPDTE can be reversibly transformed upon photoirradiation, resulting in different photophysical properties (i.e., different energy levels). Upon irradiation of the SO‐PCN MOFs at *λ*>450 nm, the open state of BPDTE was induced, and energy transfer occurred from the TCPP excited triplet state to ^3^O_2_, stimulating the generation of ^1^O_2_. However, if SO‐PCN is irradiated at λ=365 nm, BPDTE is transformed into the closed state, which is lower in energy than the TCPP, and thereby energy transfer happens to the closed BPDTE, resulting in the quenching of ^1^O_2_ generation. Therefore, the introduction of BPDTE in porphyrinic MOFs can modulate ^1^O_2_ regeneration and this strategy shows great potential for controllable PDT.

The absorption spectra of porphyrins are mostly in the visible light range, resulting in poor tissue penetration, and thereby there are limits for in vivo PDT with porphyrin‐based MOFs. Near‐infrared (NIR) light has certain advantages over visible light including deeper tissue penetration, and higher sensitivity and resolution. This makes it attractive to harvest NIR light with porphyrin‐based MOFs for NIR‐triggered PDT. Some studies demonstrated that lanthanide‐doped UCNPs can transform NIR light to visible light and thereby excite photosensitizers under NIR irradiation for enhanced PDT.^[50]^

Recently, several studies reported the combination of porphyrin‐based MOFs with UCNPs for NIR‐triggered PDT with enhanced therapeutic efficacy.[^38c^, ^38d^] Li et al. reported heterodimers (UCMOFs) that were composed of PCN‐224 and PVP‐coated lanthanide‐doped UCNPs for NIR‐triggered tumor therapy.^[38c]^ UCNPs absorbed NIR light and emitted visible light for energy transfer to TCPP in PCN‐224, resulting in ^1^O_2_ generation. The results revealed that the cytotoxic ^1^O_2_ could be generated from UCMOFs instead of PCN‐224 upon 980 nm laser irradiation. Moreover, in vivo antitumor activity tests confirmed the significant NIR‐triggered PDT ability of UCMOFs. This strategy enables the application of porphyrin‐based MOFs with NIR light harvesting functionality for NIR‐triggered PDT of large or deep tumors. He et al. also developed MOF‐UCNP core–shell nanoparticles by DNA‐mediated assembly of PCN‐224 with UCNPs.^[38d]^ Upon NIR laser irradiation, MOF‐UCNP nanoparticles could produce ^1^O_2_ and the yield increased with the loading of UCNPs. The results showed that less cell death was observed after 980 nm laser irradiation of PCN‐224 without UCNPs, but a reduction in cell viability of 63.7 % occurred after NIR irradiation of MOF‐UCNP nanoparticles. This indicates the great potential of MOF‐UCNP nanoparticles for NIR‐triggered PDT.

#### 3.1.3 Positive Targeted Photodynamic Therapy

In general, nanoparticles have a passive targeting ability for improving accumulation of their cargos at the tumor site due to the enhanced permeability and retention (EPR) effect. However, surface modification can endow nanoparticles with an active targeting ability, facilitating precise positioning at the targeted site and promoting cellular uptake. Due to abundant functional groups and metal nodes on the surface, porphyrin‐based MOFs can be easily modified by host–guest reactions, and now various porphyrin‐based MOFs have been modified with targeting moieties for enhanced PDT.[^35d^, ^36e^, ^51^]

Zhou et al. modified PCN‐224 NMOFs with folic acid (FA), of which the receptor (FAR) is overexpressed in tumor cells,^[35d]^ through the coordination between the carboxylate groups of FA and Zr_6_ clusters. The results showed that FA‐modified PCN‐224 NMOFs had better cellular uptake by FAR‐positive Hela cells than PCN‐224 NMOFs due to the FA receptor‐mediated endocytosis, and further improved PDT efficacy against Hela cells. Zhang et al. reported hyaluronic acid (HA)‐coated Zr^IV^‐based porphyrinic NMOFs (PZM) for enhanced PDT due to the CD44‐targeting of HA to CD44‐overexpressed tumor cells.^[51]^ The in vivo results confirmed that more HA‐coated PZM NMOFs were distributed in CD44‐positive tumor sites compared to PZM NMOFs, and they exhibited remarkable tumor growth inhibition.

In addition, modification of porphyrin‐based MOFs with specific biomolecules, such as cell membranes, DNA, and antibodies, also results in active targeting ability.[^38d^, ^52^] For instance, Zhang's group and Cheng's group decorated porphyrin‐based NMOFs with tumor cell membranes for immune escape and homologous targeting.[^52b^, ^52c^] Tumor cells display immune tolerance and tumor homologous binding, which are closely associated with their specific plasma membrane proteins. Interestingly, Zhao et al. established a universal method for DNA functionalization of MOFs via surface coordination chemistry, and further modified PCN‐224 NMOFs with DNA aptamers (AS1411), which endowed PCN‐224 NMOFs with specific molecular recognition capability for specific targeting of human breast cancer cells MDA‐MB‐231.^[52a]^

#### 3.1.4 Tumor Microenvironment‐Associated Photodynamic Therapy

Porphyrins show great potential for PDT, but the oxygen dependence limits the therapeutic efficacy of PDT owing to the hypoxia conditions in most tumor cells/tissues.^[53]^ Moreover, the oxygen consumption in PDT exceeds the oxygen supply by tumor blood vessels, which further aggravates hypoxia in tumor cells/tissues, and thereby decreases PDT efficacy.^[54]^ To overcome hypoxia in the tumor microenvironment, combining MOFs with other adjuvants (e.g., oxygen carriers, peroxidase, and interference agents of oxygen consumption[^51^, ^55^]) or using its own components (e.g., Cu, Fe, Mn metal ions in MOF skeletons for Fenton‐like reactions^[56]^) is an efficient strategy to increase the intratumoral O_2_ level and thereby achieve better therapeutic efficacy for PDT.

**Figure 6:**
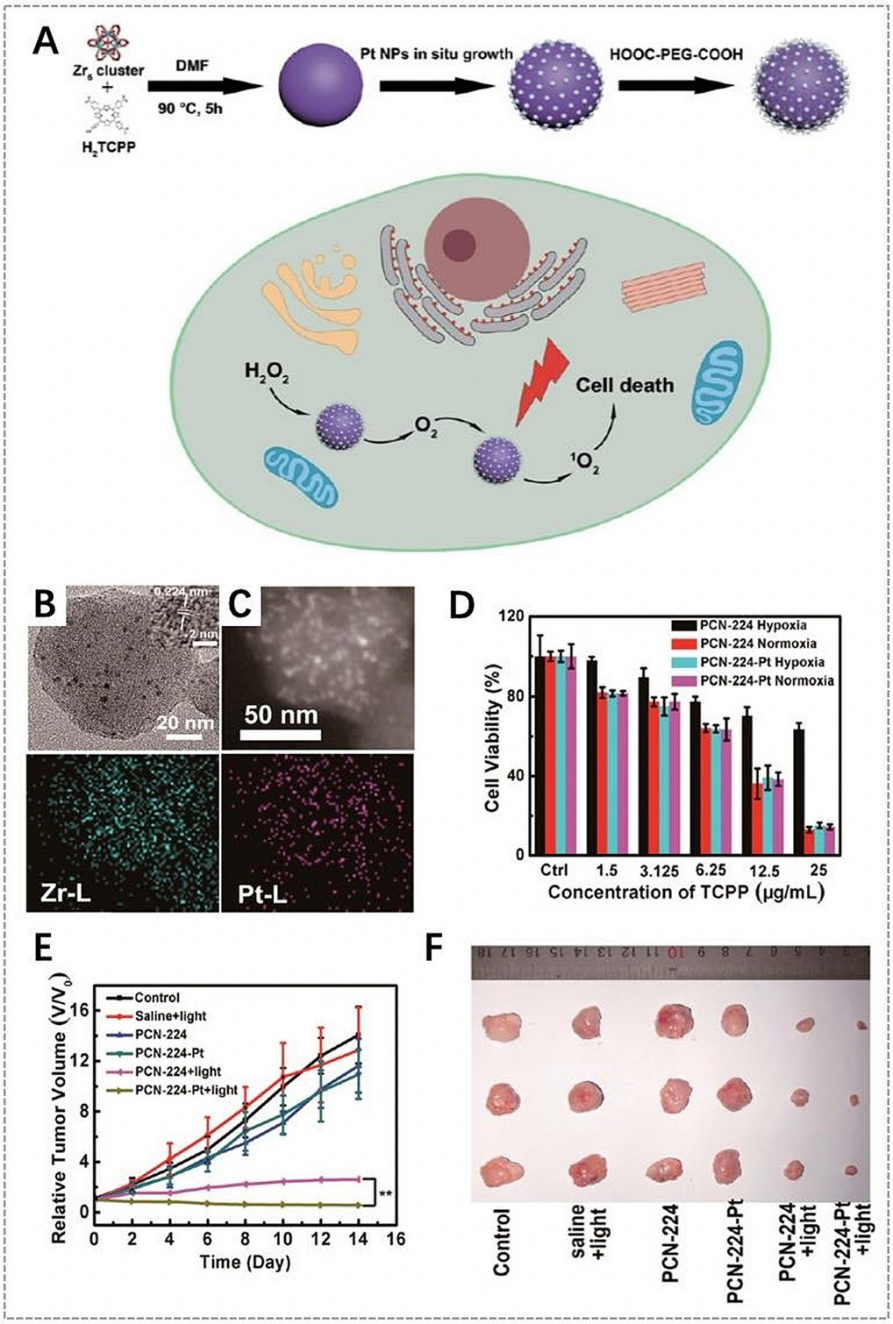
A) Synthesis of PCN‐224‐Pt NMOFs for enhanced PDT. B) Transmission electron microscopy (TEM) image of PCN‐224‐Pt NMOFs. C) Top: High‐angle annular dark‐field scanning transmission electron microscopy (HAADF‐STEM) image of PCN‐224‐Pt NMOFs; bottom: the corresponding elemental mappings of the Zr‐L edge (left) and Pt‐L edge (right) signals. D) In vitro PDT cytotoxicity of PCN‐224 and PCN‐224‐Pt under different conditions. E) Changes of relative tumor volume in vivo after various treatments. F) Photographs of the corresponding tumors after various treatments. (A–F) Reproduced with permission.^[39]^ Copyright 2018, American Chemical Society.

Ren et al. reported platinum‐decorated PCN‐224 (PCN‐224‐Pt) NMOFs for enhanced PDT (Figure 6).^[39]^ Due to the higher level of H_2_O_2_ in the tumor microenvironment, Pt nanoparticles with catalase‐like activity could catalyze the decomposition of intratumoral H_2_O_2_ to generate O_2_, which could facilitate the further generation of cytotoxic ^1^O_2_ to kill tumor cells. In vitro results showed that PCN‐224‐Pt presented much higher lethality upon 638 nm laser irradiation than that of PCN‐224 under the hypoxia conditions. The in vivo antitumor potential of PCN‐224‐Pt in an H22 tumor‐bearing mice model confirmed that tumor growth was completely inhibited after the injection of PCN‐224‐Pt and subsequent irradiation, but only partial tumor inhibition was observed for PCN‐224 after PDT. Lin et al. proposed to use 5,10,15,20‐tetra(*p*‐benzoato)porphyrins (TBP) as linkers and Fe_3_O clusters as metal nodes for the synthesis of porphyrinic NMOFs (Fe‐TBP), in which the Fe_3_O clusters could decompose the intratumoral H_2_O_2_ through the Fenton reaction to produce more O_2_ for PDT and thereby effectively circumvent cellular hypoxia.^[56a]^ Compared to free TBP and Hf‐TBP NMOFs constructed from Hf‐based clusters and TBP linkers, Fe‐TBP NMOFs showed highest PDT efficacy under both normoxic and hypoxic conditions. Obviously, Fe‐TBP NMOFs are promising for enhanced PDT in hypoxia due to the use of inherent ingredients in porphyrinic MOFs without the addition of other adjuvant agents.

In addition, the increase of the ROS level can be achieved by decreasing the glutathione (GSH) level in tumor cells, because GSH can weaken the ROS production from the photosensitizers and further decrease PDT efficacy.^[57]^ To decrease the GSH level in tumor cells, Li et al. constructed metalloporphyrinic NMOFs (MOF‐2) that assembled from Al^3+^ nodes and TCPP(Cu) linkers.^[58]^ As the active center of MOF‐2, Cu^2+^ can specifically bind and absorb GSH and thus decrease the GSH level, thereby increasing the ROS level. Compared to NMOFs without Cu^2+^ centers (MOF‐1), MOF‐2 had a GSH‐binding role and generated higher concentration of ROS. In vitro results showed that the therapeutic effect of MOF‐2 was comparable to that of the antineoplastic drug camptothecin (CPT). These results offer an interesting idea for controlling the tumor microenvironment by directly decreasing the intracellular GSH level to enhance PDT efficacy.

Specific tumor microenvironment‐associated controllable ^1^O_2_ release is also an attractive strategy for enhanced PDT.^[59]^ For instance, Tang et al. developed a bimetallic porphyrinic MOF (NP‐1), which could be activated by hydrogen sulfide (H_2_S) signaling molecules to control ^1^O_2_ release for PDT in the microenvironment of a colon adenocarcinoma tumor.^[59a]^ NP‐1 was synthesized by the self‐assembly of TCPP(Zn) linkers and Cu^2+^ nodes. In vitro results showed that NP‐1 NMOFs were activated by H_2_S in cells to induce apoptosis of tumor cells upon irradiation due to the release of Cu^2+^ from NP‐1 NMOFs. In vivo antitumor results also demonstrated that the high H_2_S levels in colon adenocarcinoma tumors significantly enhanced the antitumor efficacy owing to the H_2_S‐responsive PDT.

More recently, Jiang et al. found that 2D Cu‐TCPP MOF nanosheets exhibited the selective generation of ^1^O_2_ in the tumor microenvironment and the depletion of GSH, presenting augmented tumor therapeutic efficacy.^[60]^ Here, the TCPP linkers in the nanosheets could be peroxided in the presence of H_2_O_2_ and the acidic pH in the tumor microenvironment, and be further reduced to peroxyl radicals (ROO^.^) with the help of peroxidase‐like Cu‐TCPP MOF nanosheets and Cu^2+^ ions. ^1^O_2_ could be generated in the spontaneous recombination reaction of ROO^.^ according to the Russell mechanism. Furthermore, GSH could be depleted and converted into oxidized glutathione (GSSG) by the cycle conversion of Cu^2+^ and Cu^1+^ in Cu‐TCPP MOF nanosheets. Consequently, Cu‐TCPP MOF nanosheets selectively killed tumor cells without side effects both in vitro and in vivo. These therapeutic strategies based on the tumor microenvironment can avoid the oxygen dependence and light penetration limitations inherent in PDT, offering inspiration for other tumor treatments.

### 3.2 Synergistic Therapy of Porphyrin‐Based MOFs

To date, numerous therapy modalities based on nanoplatforms, including chemotherapy, PDT, photothermal therapy (PTT), radiotherapy (RT), immunotherapy, and gas therapy, have been developed as effective techniques for treating malignant tumors.[^59c^, ^61^] However, single therapy modality often fails to achieve ideal therapeutic efficacy due to limitations, such as multidrug resistance, oxygen dependence, nonspecific heating, and serious side effects. Hence, much effort has been devoted to developing multifunctional nanoplatforms, which integrate two or more therapy modalities into a single system. Porphyrin‐based MOFs exhibit excellent PDT efficacy due to the photophysical properties of porphyrins, and porous structures endow them with controlled drug delivery. Furthermore, porphyrin‐based MOFs can integrate other functional components to obtain multifunctionality. Hence porphyrin‐based MOFs are able to combine two or more efficient therapy modalities for tumor therapy that can achieve synergistic effects for maximizing therapeutic efficacy.

#### 3.2.1 Synergistic Chemo‐ and Photodynamic Therapy

The combination of PDT and chemotherapy is an efficient dual‐modality therapy approach that has shown enhanced therapeutic efficacy and negligible toxicity.^[62]^ Here, PDT can generate toxic ROS in tumor cells/tissues that induce oxidative damage to intracellular protein and DNA, making the tumor cells highly sensitive to toxic chemotherapeutic drugs.[^56b^, ^63^] Thus, the use of lower doses of chemotherapeutic drugs could bring the desired antitumor effect with reduced toxicity for normal cells/tissues. The open porosity, stable structure, and low cytotoxicity of porphyrin‐based MOFs make them suitable drug delivery vehicles for encapsulating chemotherapeutics, and the intrinsic nature of porphyrin‐based MOFs supports satisfactory drug‐release behavior to enhance therapeutic efficacy.^[64]^ Hence, drug‐loaded porphyrin‐based MOFs show great potential for synergistic chemo‐ and photodynamic therapy.

Yin et al. reported biocompatible zirconium porphyrinic NMOFs (NPMOFs) for synergistic chemotherapy and PDT.^[37a]^ NPMOFs showed a doxorubicin (DOX) loading efficiency as high as 109 % (w/w), which was attributed to the noncovalent interactions between DOX and NPMOFs, such as π–π stacking effects, hydrophobic interactions, and electrostatic interactions. Drug release profiles showed a very slow DOX release rate in a normal biological environment (only 10.3 % of DOX released at pH 7.0 after 72 h), but a fast DOX release in the tumor microenvironment (58.5 % of DOX released at pH 5.0 after 72 h). This drug release behavior is beneficial for high therapeutic efficacy against tumor cells with few side effects for normal cells. At the same time, the high porphyrin content of NPMOFs (59.8 %) resulted in excellent PDT efficacy. A tumor cell apoptosis rate of 90 % was observed upon synergistic therapy with DOX@NPMOFs upon 655 nm laser irradiation. In vivo therapy for HepG2 tumor‐bearing mice achieved an enhanced therapeutic efficacy for DOX@NPMOFs compared to chemotherapy or PDT alone, indicating that biocompatible porphyrin‐based MOFs are highly promising for synergistic chemotherapy and PDT.

**Figure 7:**
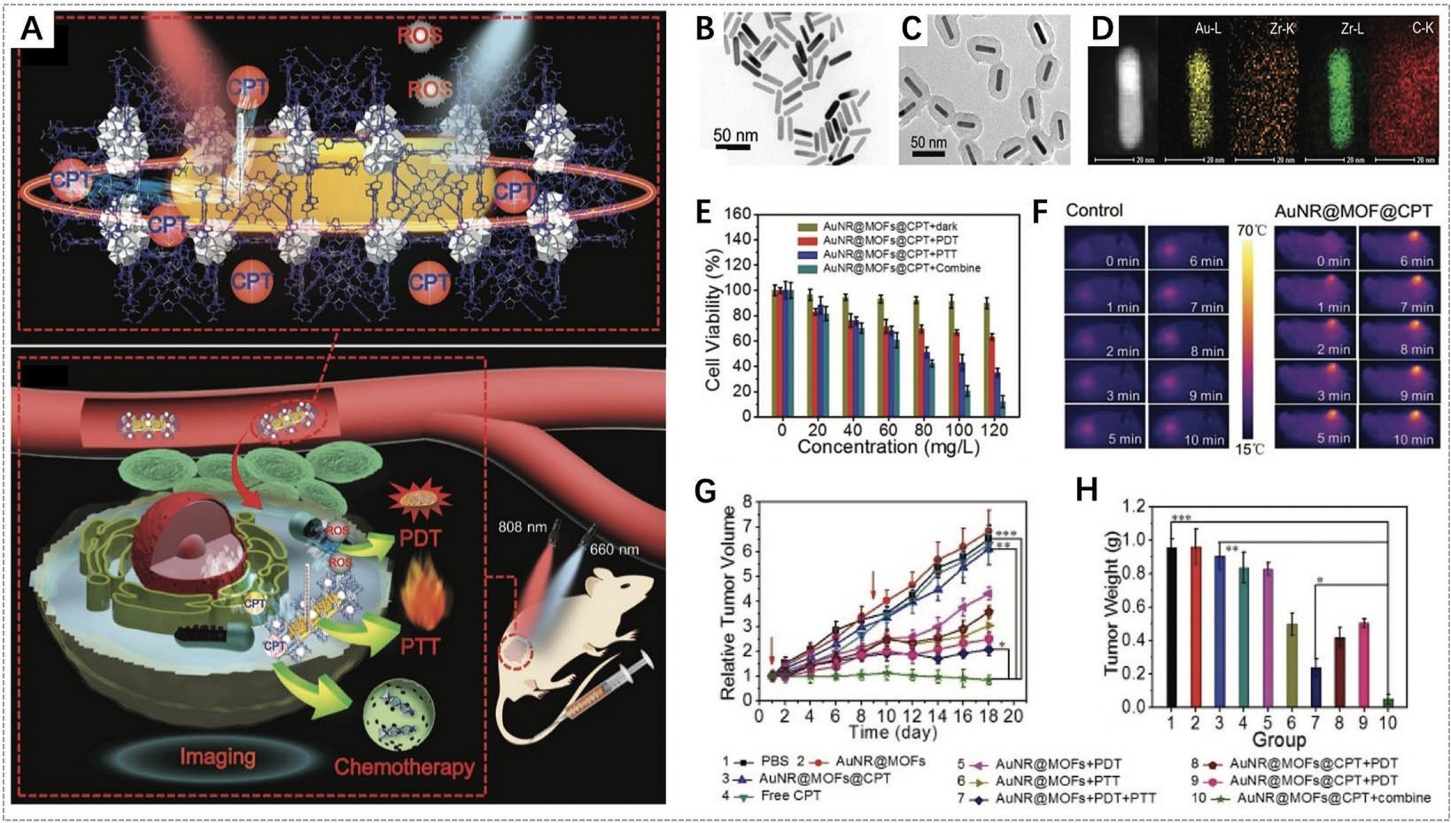
A) Illustration of AuNR@MOFs for synergistic chemotherapy and phototherapy. TEM images of B) AuNR and C) AuNR@MOFs. D) HAADF‐STEM image of AuNR@MOFs and the corresponding elemental mappings. E) In vitro cytotoxicity of AuNR@MOFs@CPT with different treatments. F) Photothermal images of the mice treated with PBS and AuNR@MOFs@CPT, respectively, upon 808 nm laser irradiation. G) Changes of relative tumor volume in vivo after various treatments (red arrows refer to the time points of treatment). H) The tumor weights of different groups after treatment for 18 days. (A–H) Reproduced with permission.^[38a]^ Copyright 2018, Wiley‐VCH.

For light‐controlled release of toxic chemotherapeutic drugs and enhanced PDT, Zhang et al. developed core–shell composite nanoparticles (AuNR@MOFs) by the growth of porphyrinic MOFs on gold nanorods (AuNR) (Figure 7).^[38a]^ These nanoparticles were used for synergistic chemotherapy and phototherapy due to the well‐defined pore structure as the drug carrier, TCPP as the photosensitizer, and AuNR as the photothermal agent. With camptothecin (CPT) as a model drug, AuNR@MOFs achieved controllable CPT release upon 808 nm laser irradiation. On the other hand, AuNR@MOFs exhibited an enhanced ability to produce ^1^O_2_ compared to single porphyrinic NMOFs upon 660 nm laser irradiation due to the enhanced light absorption and strong electromagnetic field effect of the AuNR surface. Consequently, AuNR@MOFs with NIR‐triggered drug release and phototherapy showed significant synergistic efficacy for killing tumor cells in vitro and inhibiting tumor growth and metastasis in vivo.

#### 3.2.2 Synergistic Photothermal and Photodynamic Therapy

Similar to PDT, PTT is also a targeted and local therapeutic method with minimal invasiveness and high efficacy, which uses photothermal agents to generate heat upon NIR irradiation to ablate tumor cells and tissues.^[41]^ In contrast to PDT, PTT is an oxygen‐independent and ROS‐free process mediated by photothermal agents upon NIR irradiation. Hence, the combination of PDT and PTT for tumor treatment could synergistically enhance the therapeutic effect and reduce side effects.^[65]^ On the one hand, the heat generated by PTT can improve blood flow as well as oxygen supply, and thereby intensify the sensitivity of tumor cells to oxygen‐dependent PDT; on the other hand, the ROS produced by PDT can interfere with tumor physiology and change the microenvironment, and thereby increase the heat sensitivity of the tumor cells.^[66]^

Recently, several studies on porphyrin‐based MOFs for synergistic PDT and PTT have been reported. Zhang et al. proposed to assemble porphyrinic MOFs on polydopamine (PDA) nanoparticles to form core–shell nanoparticles (PDA@MOF),^[38g]^ and the photodynamic activity of porphyrinic MOFs and the photothermal effect of PDA nanoparticles could contribute to PDT and PTT for tumors. The results indicated that PDA@MOF nanoparticles efficiently generated intracellular ROS upon irradiation with 630 nm laser light, and had excellent photothermal conversion upon irradiation with 808 nm laser light. Only 18 % of the cells were viable after incubation with PDA@MOF nanoparticles and irradiation with 630 nm and 808 nm laser light. This was much lower than the viability following single laser irradiation, suggesting synergistic tumor inhibition.

Besides composite porphyrinic NMOFs, porphyrinic NMOFs without the integration of other PTT agents also have been developed for synergistic PTT and PDT, which shorten the synthesis procedures and improve biosafety. For example, Yang et al. reported novel multifunctional metalloporphyrinic NMOF (Zr‐FeP) shuttles for PDT and low‐temperature PTT synergistic treatment.^[66]^ Zr‐FeP NMOFs were assembled from Zr_6_ clusters and TBP(Fe) metalloporphyrins and loaded with an inhibitor of heat shock protein 70 (Hsp70). The results demonstrated that Zr‐FeP NMOFs catalyzed the generation of abundant ^.^OH from endogenous H_2_O_2_ due to Fenton effect of the ion centers, and produced toxic ^1^O_2_ upon laser irradiation. Furthermore, Zr‐FeP NMOFs showed an obvious photothermal effect and the photothermal conversion efficiency was as high as 33.7 %. Consequently, upon 635 nm laser light irradiation, the Hsp70‐loaded Zr‐FeP NMOFs efficiently suppressed the tumor growth by synergistic PDT and low‐temperature PTT.

Recently, Li et al. proposed using Cu‐TCPP MOF nanosheets as phototherapy nanosystems for synergistic PTT and PDT.^[67]^ The d–d energy band transition of Cu^2+^ and the thickness of the ultrathin Cu‐TCPP MOF nanosheets led to strong NIR absorption and excellent photothermal performance. The photothermal conversion efficiency of Cu‐TCPP MOF nanosheets at 808 nm irradiation was estimated to be 36.8 % and ^1^O_2_ could be generated upon 660 nm irradiation, which induced efficient synergistic PTT and PDT in vitro and in vivo.

#### 3.2.3 Synergistic Immuno‐ and Photodynamic Therapy

Immunotherapy has an important status in tumor therapy since it is possible to stimulate an individual's own immune system to recognize and kill primary tumor cells as well as inhibit metastatic tumor cells.^[68]^ Checkpoint blockade immunotherapy, which adopts antibodies or small molecules to block negative immune regulatory pathways for priming antitumor immune, has been successfully used in clinic. Studies have demonstrated that tumor cells themselves can induce T cell apoptosis via the programmed cell death receptor 1 (PD‐1)/programmed death 1 ligand (PD‐L1) pathway for immune evasion. Consequently, the PD‐1/PD‐L1 checkpoint blockade strategy is proposed to enhance antitumor immunity by inhibiting cytotoxic T lymphocyte exhaustion. However, this type of immunotherapy elicits limited rates of systemic antitumor response for many types of tumors owing to insufficient activation of the host immune systems.^[69]^ Interestingly, PDT can excite adaptive immune responses through the release of inflammatory mediators and cytokines into the intratumoral environment. Therefore, the combination of checkpoint blockade immunotherapy and PDT can not only promote systematic immune response to inhibit the metastatic tumors, but also induce synergistic therapy for treating primary tumors. Recently, there have been some reports describing porphyrinic MOFs for synergistic immunotherapy and PDT due to easy construction of porphyrinic MOFs‐based nanoplatforms.

**Figure 8:**
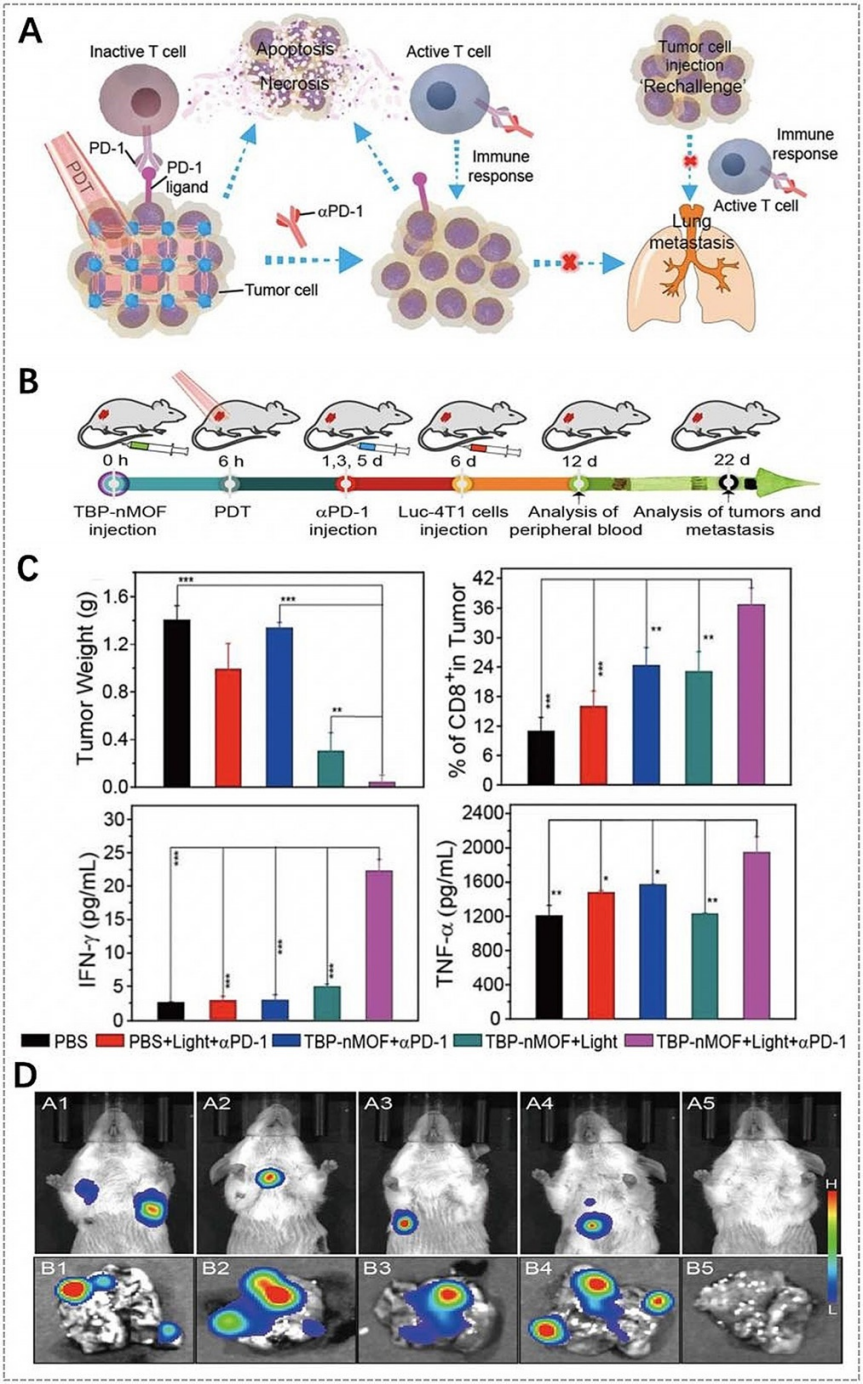
A) The proposed mechanism of antitumor immune responses induced by the combination of PDT and α‐PD‐1. B) Synergistic therapy using TBP‐nMOFs. C) Comparative analysis after various treatments (from left to right): the tumor weight after 22 days, the percentage of CD8^+^ cells, the generation of IFN‐γ and TNF‐α in mice sera obtained on the 12th day. D) Bioluminescence imaging of the mice and the lung metastatic sites of the luciferase‐4T1 (luc‐4T1) tumors corresponding to the above five treatments. (A–D) Reproduced with permission.^[70]^ Copyright 2018, American Chemical Society.

Zhang et al. reported benzoporphyrinic NMOFs (TBP‐NMOFs) for PDT, and further investigated synergistic PDT and α‐PD‐1 checkpoint blockade immunotherapy (Figure 8).^[70]^ TBP‐NMOFs, which were assembled by the coordination of tetrabenzoporphyrin linkers with 10‐connected Zr_6_ clusters, exhibited efficient ^1^O_2_ generation in the hypoxic microenvironment for PDT. The results demonstrated that TBP‐NMOF‐induced PDT could not only kill the 4T1 murine breast tumor cells, but also augment the presentation of tumor‐activated antigens, further stimulating adaptive immune response for initiating the secretion of inflammatory cytokines (IFN‐γ, TNF‐α) and recruiting tumor‐infiltrating T cells (CD4^+^, CD8^+^). Further, they investigated the antitumor effect of low O_2_‐dependent PDT and α‐PD‐1 in vivo. When PD‐1 antibodies were injected, TBP‐NMOF‐induced PDT dramatically inhibited the growth of 4T1 tumors without body weight loss. Furthermore, the combination therapy was more efficient than PDT alone in preventing tumor recurrence and remarkably prevented lung metastasis of 4T1 tumors for 22 days. Similarly, Lin et al. also utilized porphyrinic MOFs (Fe‐TBP) and α‐PD‐L1 for synergistic PDT and immunotherapy.^[56a]^ It is interesting that the Fe_3_O clusters in Fe‐TBP NMOFs could overcome hypoxia by the Fenton reaction and enhance immunogenic PDT for priming tumor immunotherapy.

Obviously, the use of porphyrinic MOFs for synergistic immuno‐ and photodynamic therapy has shown great potential for the therapy of many difficult‐to‐treat tumors and inhibition of metastatic tumor cells, because immunogenic PDT can prime tumor immunotherapy to promote the response rates, and thereby significantly promote immunotherapeutic efficacy. In addition to checkpoint blockade immunotherapy based on antibodies or small‐molecule agents, it can be imagined that the use of porphyrinic MOF platforms to combine PDT with other immunotherapeutic strategies would be attractive.^[71]^

#### 3.2.4 Other Synergistic Therapies

Radiotherapy (RT), which uses ionizing irradiation such as X‐rays to destroy localized solid tumors, is a common therapy modality in clinic. Numerous nanoplatforms containing high‐Z elements, which are able to interact with ionizing radiation to produce photo/auger electrons and then produce reactive free radicals to destroy tumor cells, have been applied for RT of tumors.^[72]^ Biocompatible and biodegradable porphyrinic MOFs are formed by the coordination of porphyrin linkers and metal nodes, which is ideal to introduce high‐Z metal elements into MOFs for use in synergistic PDT and RT. Liu et al. reported Hf‐TCPP NMOFs assembled from Hf^4+^ and TCPP through a solvothermal process,^[44]^ in which TCPP linkers exhibited good PDT ability. The Hf^4+^ metal centers, as a high‐Z element, served as a radio‐sensitizer to enhance RT. Due to the ISC enhanced by heavy Hf^4+^ nodes, Hf‐TCPP NMOFs were extremely effective in producing ^1^O_2_ upon 661 nm irradiation for PDT. In vivo results on 4T1 breast tumor‐bearing mice showed that synergistic PDT and RT with Hf‐TCPP NMOFs greatly inhibited tumor growth and had enhanced therapeutic efficacy compared to PDT or RT alone.

**Figure 9:**
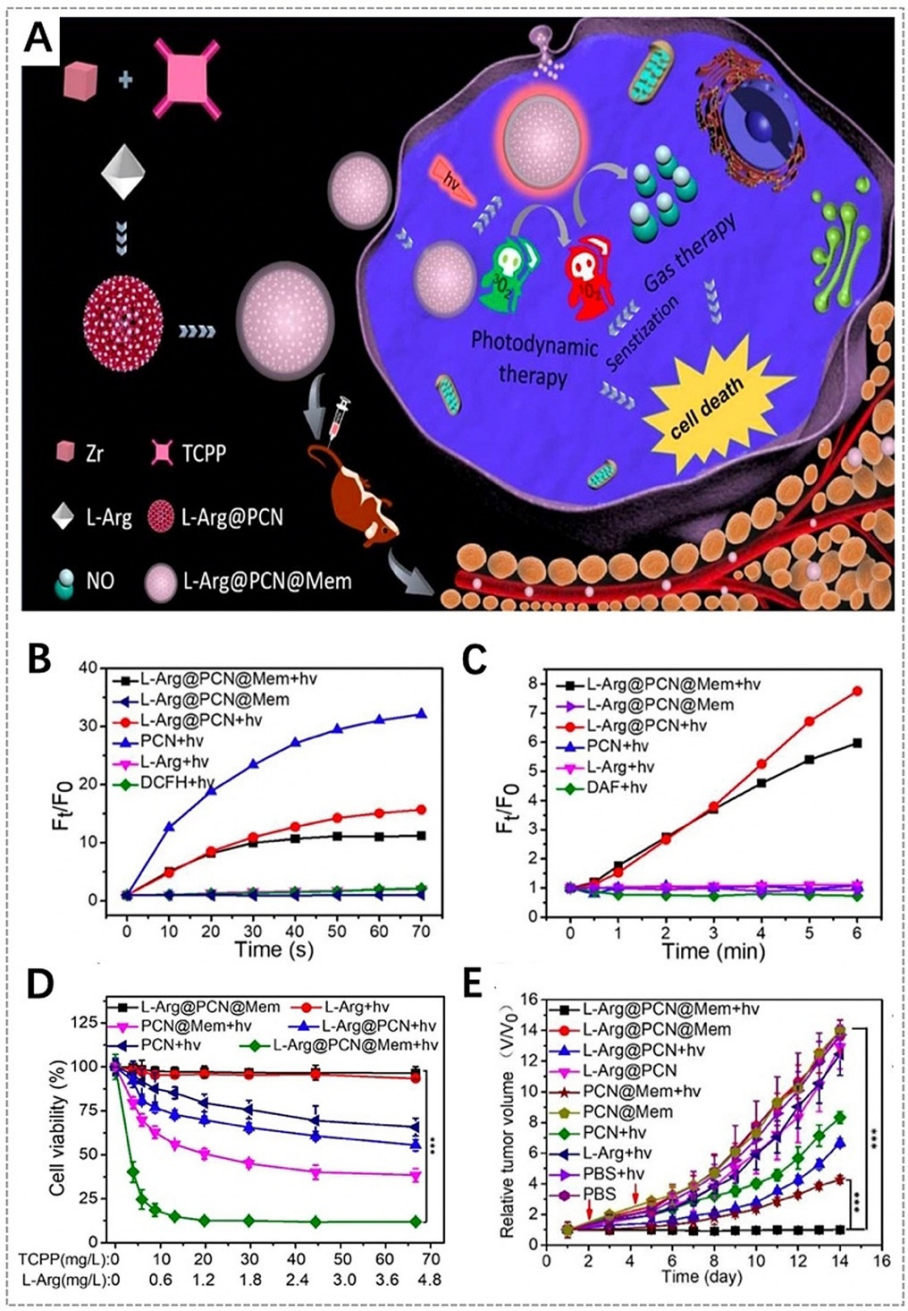
A) Illustration of l‐Arg‐incorporated PCN‐224 NMOFs for combined gas therapy and PDT. B) ROS generation in different groups detected by a 2′,7′‐dichlorofluorescin diacetate (DCFH‐DA) probe. C) NO generation in different groups. D) In vitro cytotoxicity of 4T1 cells after different treatments. E) Changes of relative tumor volume in vivo after various treatments (red arrows refer to the light irradiation time points). (A–E) Reproduced with permission.^[52c]^ Copyright 2018, Elsevier Ltd.

Gas therapy also has been developed for tumor therapy due to its negligible side effects and enhanced therapeutic efficacy. Various gaseous transmitters, such as hydrogen sulfide (H_2_S), nitric oxide (NO), and carbon monoxide (CO), can be utilized for gas therapy, which can accelerate tumor cell apoptosis, prevent tumor cell proliferation, and selectively protect the activity and physiological function of normal cells.^[73]^ Recently, Cheng et al. reported synergistic NO gas therapy and PDT to treat tumors based on l‐arginine (l‐Arg)‐incorporated PCN‐224 NMOFs (Figure 9).^[52c]^ Here, l‐Arg, a natural NO donor, could release NO for gas therapy via oxidization through ROS, which were provided by PDT with PCN‐224 upon 660 nm irradiation. An in vitro MTT assay showed that the viability of 4T1 cells treated with l‐Arg‐incorporated PCN‐224 was lower than that of cells treated with PCN‐224 upon 660 nm irradiation. Of special note, a cell apoptosis assay executed by flow cytometry showed that l‐Arg‐incorporated PCN‐224 was much more effective in PDT under hypoxic conditions than pure PCN‐224, proving that NO can sensitize PDT in hypoxic tumors. Furthermore, an in vivo antitumor study showed that the therapeutic efficacy of the PCN‐224 was weaker than that of the l‐Arg‐incorporated PCN‐224 upon irradiation with 660 nm light, indicating that synergistic PDT and gas therapy enhanced antitumor effects to a certain extent. This type of nanoplatform with gas therapy and PDT provides a novel therapy paradigm to overcome the hypoxia barrier for efficient tumor treatment.

Hydrogen gas as a clean energy source has recently been discovered to have curative effects in many diseases, and it possesses a huge potential in tumor treatment due to its high biosafety and satisfactory therapeutic effect. He et al. developed a nanoscale Pd‐MOF, which was assembled from Pd^2+^ nodes and TPyP porphyrin linkers, for hydrogenothermal therapy of tumors.^[74]^ Due to the strong hydrogen‐binding and catalytic hydrogenation capacity of Pd, Pd‐MOF could be used to deliver highly reductive hydrogen gas. The results showed that Pd‐MOF displayed a high hydrogen loading capacity and continuous hydrogen release profile. In photothermal experiments Pd‐MOF loaded with hydrogen gas showed higher photothermal conversion efficiency (44.2 %) than Pd‐MOF alone (27.7 %) at 808 nm irradiation. Further in vitro and in vivo results proved the synergistic antitumor effects of hydrogen gas and PTT.

Selectively cutting off the nutrient supply and metabolic pathways of tumor cells is another efficient strategy for tumor therapy. Zhang et al. designed a tumor‐targeted cascade bioreactor mem@catalase@GOx@PCN‐224 (mCGP) by the encapsulation of glucose oxidase (GOx) and catalase in tumor cell membrane‐camouflaged PCN‐224 NMOFs for synergistic starvation and PDT.^[52b]^ It was found that mCGP could facilitate microenvironmental oxygenation by catalyzing endogenous H_2_O_2_ to generate O_2_ by catalase, which would subsequently accelerate the consumption of intracellular glucose by GOx and increase the generation of ^1^O_2_ under 660 nm irradiation. This could cut off tumor glucose supply and disturb glucose metabolism related cellular elements for starvation therapy as well as overcome the hypoxia issue for enhanced PDT. In vivo experiments revealed that mCGP possessed a certain antitumor activity without 660 nm irradiation for long‐term starvation effects. On the other hand, mCGD showed a complete suppression of 4T1 tumors in the presence of 660 nm irradiation without obvious side effects, which might be attributed to the significant synergistic effects of PDT and long‐term tumor starvation therapy.

### 3.3 Imaging‐Guided Therapy of Porphyrin‐Based MOFs

Nanomaterials have been comprehensively studied as novel imaging agents or functional platforms for bio‐imaging, which produce signals or enhance signal contrast at specific tissues. Bio‐imaging enables the visualization, evaluation, and quantification of biological processes at the molecular and cellular levels in a noninvasive means, and shows great potential in the earlier diagnosis of diseases, on‐going assessment of treatments, and optimization of disease therapeutic protocols.^[23b]^ Therefore, the construction of nanosystems for imaging‐guided therapy by the integration of therapy modalities and bio‐imaging techniques is promising to maximize the therapeutic efficacy in a time‐ and position‐resolved manner.^[75]^ Over the years, porphyrin‐based MOFs have been utilized as promising fluorescence imaging‐guided therapy nanosystems owing to their excellent fluorescence and therapeutic properties. In addition, imaging modes of porphyrin‐based MOFs can be branched to magnetic resonance imaging (MRI), X‐ray computed tomography (CT), photoacoustic imaging (PAI), and photothermal imaging (PTI) by employing metal nodes in MOFs or metal centers in porphyrin rings, or by incorporating other contrast agents in MOFs.

#### 3.3.1 Fluorescence Imaging‐Guided Therapy

Fluorescence imaging has been a powerful imaging mode for both in vitro and in vivo imaging because it is noninvasive and offers high signal sensitivity. Fluorescence imaging relies on the properties of fluorophores that absorb the photon energy in a certain band of wavelengths and then emit new photon energy in a band of longer wavelengths. Fluorescence imaging is rapid and suitable for high‐throughput screening.^[76]^ Porphyrins as popular fluorophores have been incorporated into MOFs for both in vitro and in vivo fluorescence imaging.[^52a^, ^77^] The use of fluorescence imaging to guide therapy allows the visualization of the distribution and evolution of the tumors/particles during treatment, and thereby ensures selectively controllable therapy with high efficacy and safety. Fluorescence imaging‐guided therapy nanosystems based on porphyrin‐based MOFs can be easily constructed for efficient therapy due to the combination of fluorescence and photosensitivity from porphyrins.

**Figure 10:**
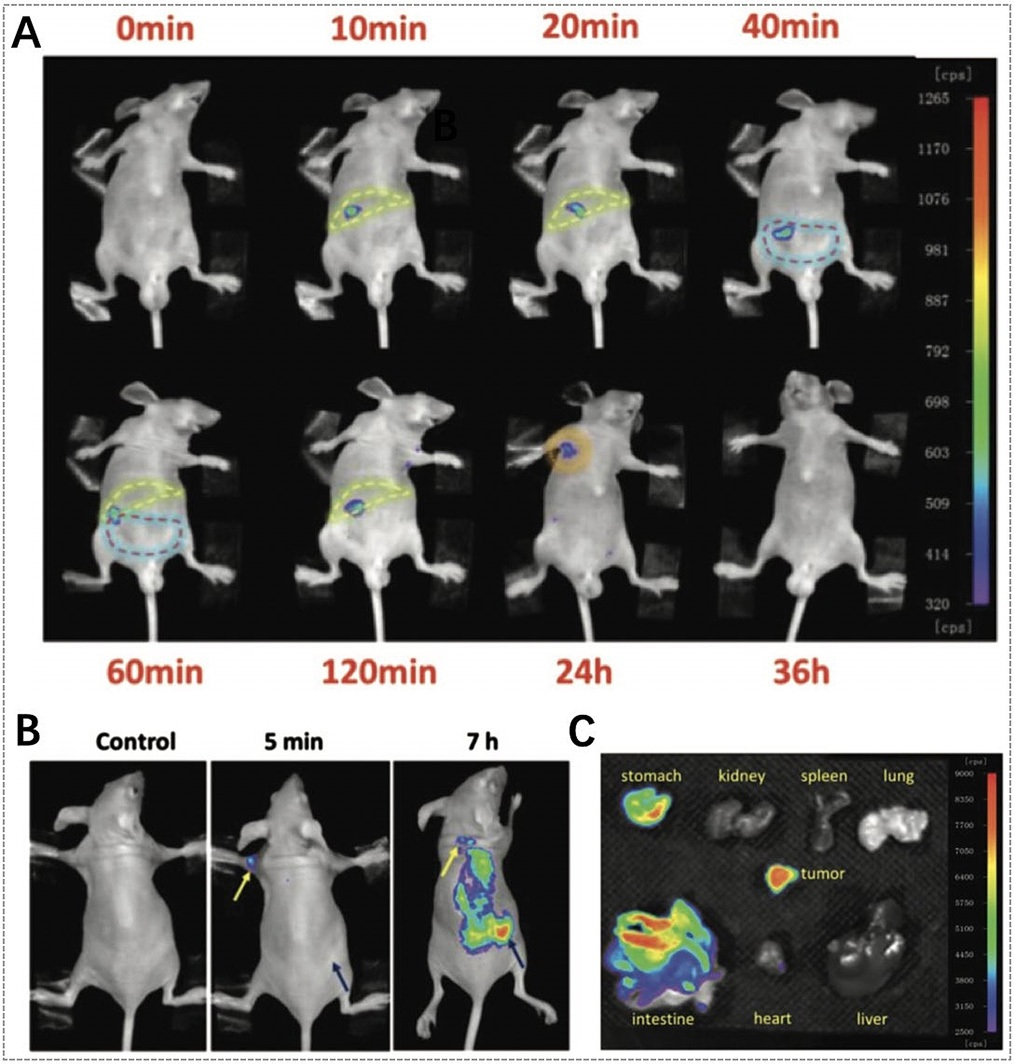
Fluorescence images of A) the mouse (yellow dotted lines refer to the liver region, red dotted lines refer to the intestine region, and green dotted lines refer to the lymph node) and B) the tumor‐bearing mouse (yellow arrows refer to the small lymph node, blue arrows refer to the subcutaneous transplantable tumor) recorded at excitation of 530 nm and emission of 700 nm after injection of NPMOFs. C) Fluorescence images of dissected organs of a tumor‐bearing mouse. (A–C) Reproduced with permission.^[37a]^ Copyright 2017, Wiley‐VCH.

In 2017, Yin et al. developed the first biocompatible porphyrinic NMOFs for fluorescence imaging‐guided tumor therapy (Figure 10).^[37a]^ Porphyrinic NMOFs (NPMOFs) with a high TCPP content (59.8 %) achieved efficient fluorescence imaging. The results showed that the fluorescence of TCPP was observed at 651 nm with a weaker shoulder at 710 nm when excited at 514 nm, but only one emission peak at 689 nm for NPMOFs.^[78]^ The strong red emission and long Stokes shift of NPMOFs resulted in a high signal‐to‐noise ratio and resolution in fluorescence imaging due to the low excitation interference, autofluorescence, and scattering light from the biological tissue. In a mouse model, the absorption, distribution, metabolism, and excretion (ADME) processes of NPMOFs were observed by tracking the fluorescent trajectory, indicating the biocompatibility of NPMOFs in mammals. Furthermore, in HepG2 tumor‐bearing mice, the in vivo fluorescence of NPMOFs clearly confirmed the distributions of NPMOFs in tumors with high resolution and signal‐to‐noise ratio, suggesting that porphyrinic NMOFs are promising as fluorescence imaging‐guided therapy platforms for earlier tumor diagnosis and enhanced therapy. On the other hand, composite porphyrinic NMOFs have also been constructed for fluorescence imaging‐guided therapy.[^38a^, ^79^] For example, Zhang et al. assembled porphyrinic NMOFs on AuNRs to form core–shell nanocomposites for fluorescence imaging‐guided PDT, PTT, and chemotherapy.^[38a]^ The introduction of porphyrinic NMOFs made the nanocomposites suitable for PDT, chemotherapy, and fluorescence imaging, while AuNRs exhibited excellent PTT properties.

In addition, fluorescence imaging‐guided therapy can also be used for real‐time monitoring and assessment of the therapeutic efficacy according to the changes of fluorescence signals, and can be used to further improve the therapeutic efficacy and avoid under‐ or overtreatment. Lei et al. designed a photosensitive and caspase‐responsive porphyrin@MOF nanoprobe, which was assembled from porphyrin (TMPyP), folate targeting‐motif, and a caspase‐sensitive fluorescent dye, i.e., Cy3‐labeled caspase‐3 substrate peptide, in MOFs.^[45]^ Here, the fluorescence of Cy3 was quenched until the activated caspase‐3 specifically cleaved the peptide and Cy3 was released from the MOF for fluorescence imaging, resulting in a caspase‐responsive sensing strategy for monitoring cell apoptosis. These results demonstrated that biocompatible TMPyP@MOFs provided enhanced ^1^O_2_ yield and folate targeting tumor therapy as well as the ability for in situ imaging of therapeutic efficacy via caspase‐3 activation. The switch‐on signal provides an effective way to image intracellular caspase activity for real‐time monitoring and evaluation of treatment effects.

#### 3.3.2 MRI‐Guided Therapy

MRI is another noninvasive medical method to obtain images of the human anatomy and physiological processes with high spatial resolution. It utilizes an external magnetic field to detect radiofrequency signals produced by protons, typically the hydrogen atoms in water from fat and tissues. The acquired signals can be utilized to compare different tissues, distinguish lesions from healthy tissues, and construct anatomical maps of the human body.^[80]^ Compared to fluorescence imaging, MRI possesses the advantages of better spatial resolution, higher soft tissue contrast, and unlimited penetration depth, but exhibits the disadvantage of low sensitivity. MRI contrast agents are typically used to reduce proton relaxation times and further improve the image quality. For porphyrin‐based MOFs, the metal nodes of the MOF skeleton, the chelation of paramagnetic metal ions in porphyrin rings, and the incorporation of other contrast agents in MOFs make MRI‐guided therapy possible.

Recently, Wang et al. reported that gadolinium porphyrinic NMOF sheets (Gd‐TCPP) displayed a high relaxation rate (40.8 mm ^−1^ s^−1^) and ^1^O_2_ production upon 660 nm irradiation due to the high Gd^3+^ content in the MOFs and the TCPP photosensitizer.^[33d]^ Therefore, Gd‐TCPP nanosheets are potent for *T*_1_‐weighted MRI‐guided PDT. Yin et al. proposed to bind paramagnetic metal ions into porphyrin rings by chelation to develop metalloporphyrinic MOFs for *T*_1_‐weighted MRI‐guided therapy.^[81]^ Through the chelation of paramagnetic Mn ions in porphyrin rings, metalloporphyrinic MOFs possessed excellent *T*_1_‐weighted MR contrast capacity and high photothermal conversion and heat‐responsive NO release. In vivo MRI experiments showed that metalloporphyrinic MOFs were efficiently accumulated at the tumor site after intravenous injection into mice, and tumor growth was completely inhibited when exposed to 808 nm laser for PTT and NO gas therapy, indicating the potential for MRI‐guided therapy.

#### 3.3.3 Multimode Imaging‐Guided Therapy

Single‐mode bio‐imaging is usually insufficient for comprehensive imaging and cannot provide the complete characterization needed to diagnose diseases due to its inherent limitations, such as limited signal sensitivity, tissue penetration depth, and spatial resolution. To overcome these problems, various studies have concentrated on incorporating multimode imaging properties in a single MOF‐based system.^[82]^ For porphyrinic MOFs, the use of functional components of the MOFs themselves or the assembly of other imaging agents can achieve multimode imaging‐guided therapy, which could avoid inherent limitations of single‐mode imaging‐guided therapy and provide more accurate localization of lesions/particles as well as guidelines of therapy.

**Figure 11:**
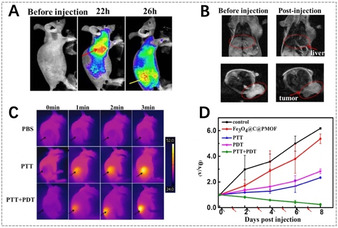
A) Fluorescence images of the tumor‐bearing mouse before and after injection of Fe_3_O_4_@C@PMOF (red arrow refers to the liver region, yellow arrow refers to the tumor region). B) *T*_2_‐weighted MRI of the tumor‐bearing mouse (upper red dot lines refer to the liver region, lower red dot lines refer to the tumor region). C) Infrared thermal photographs of tumor‐bearing mice in different groups (black arrows refer to the tumor region). D) Changes of relative tumor volume in vivo after various treatments (black and red arrows refer to the injection and irradiation time points, respectively). (A–D) Reproduced with permission.^[79]^ Copyright 2017, Nature Publishing Group.

For example, Yin et al. developed a biocompatible nanocomposite (Fe_3_O_4_@C@PMOF) with fluorescence imaging and MRI functions for dual‐mode imaging‐guided phototherapy (Figure 11).^[79]^ Specifically, Fe_3_O_4_@C cores were selected as *T*_2_‐weighted MRI contrast agents and photothermal agents, and porphyrinic MOF shells had fluorescence imaging and PDT abilities. Such dual‐mode imaging nanoplatforms achieved high sensitivity of fluorescence imaging as well as deep penetration and great spatial resolution of MRI, and more precise in vivo information could be provided for enhancing the safety and therapeutic efficacy. Following intravenous injection of Fe_3_O_4_@C@PMOF nanocomposites in MCF‐7 tumor‐bearing mice, 22 h later the fluorescence imaging showed that nanocomposites were primarily localized in the liver region rather than in other organs. The tumor area slowly brightened and became the brightest site in the mice after 26 h, indicating that the accumulation of nanocomposites at the tumor site had gradually increased. The results were also confirmed by dramatic dimming at the tumor site in *T*_2_‐weighted MR imaging. Owing to their high tumor accumulation, Fe_3_O_4_@C@PMOF nanocomposites were used for imaging‐guided phototherapy; an obvious PTT/PDT synergetic effect was observed for MCF‐7 breast tumor but the toxicity for normal tissues was low.

Clearly, it is also valuable to develop porphyrinic MOFs without additional components for multimode imaging‐guided therapy due to its simplicity and efficiency. Wu et al. reported ultrathin 2D Cu‐TCPP MOF nanosheets for dual‐mode imaging‐guided phototherapy.^[67]^ The Cu^2+^ nodes in Cu‐TCPP nanosheets offered excellent photothermal properties for PTT, PTI, and *T*_1_‐weighted MRI. Meanwhile, porphyrin TCPP provided the ability to produce ^1^O_2_ for PDT. The results indicated that Cu‐TCPP nanosheets have tremendous potential for synergistic PTT and PDT, guided by PTI and MRI.

CT is an important medical imaging technique, which is based on the attenuation of X‐rays by a specimen resulting in 3D images with high spatial resolution.^[83]^ The high‐Z number elements in MOFs can be chosen as CT contrast agents. In addition, PAI is a recently developed imaging technique that integrates optical excitation and ultrasound detection.^[84]^ This technique displays high selectivity and deep penetration, and can be used to acquire high‐resolution and strong‐contrast tissue images. To combine the merits of PTI, CT, and PAI, Yang et al. developed multifunctional metalloporphyrinic NMOF (Zr‐FeP) shuttles as an “all‐in‐one” nanoplatform to realize trimode imaging‐guided therapy.^[66]^ In vivo PAI experiments demonstrated that a much stronger PA signal was generated in the tumor tissue 2 h after tail vein injection of nanoshuttles into mice, indicating the considerable accumulation of nanoshuttles in the tumor. Zr‐FeP MOF nanoshuttles exhibited good CT capability due to the high‐Z component for CT imaging. Additionally, the outstanding photothermal performance of Zr‐FeP MOF nanoshuttles endowed remarkable PTI ability in vivo, and after treatment with Zr‐FeP MOF nanoshuttles the tumor site temperature rapidly increased by 17 °C upon exposure to 635 nm laser for 5 min. Therefore, the fascinating trimode imaging capability of Zr‐FeP MOF nanoshuttles would be a powerful tool for precise tumor diagnosis and imaging‐guided therapy.

### 3.4 Biosensing

A large number of physiological species play a key role in regulating cellular functions and physiological activities, of which the changes in the concentrations and types indicate disruptions or pathological changes in physiological environments. Therefore, specific and sensitive biosensing systems have been increasingly used to detect and monitor the variations for disease diagnosis and biological processes in living organisms. Porphyrins and their derivatives have been widely studied as small‐molecule biosensors, due to their excellent physicochemical characteristics that can be used for signal interaction with host molecules. Moreover, porphyrins can mimic many biological functions. For example, porphyrins can reversibly bind with gaseous compounds and can undergo photophysical and/or redox processes mediated by the target analytes.^[8a]^ Interestingly, hemin, which is also called iron protoporphyrin IX, has been utilized for biosensing, because it can act not only as a redox mediator based on the electrochemical activity of the Fe^III^/Fe^II^ reversible redox pair, but also as a catalyst based on its peroxidase‐like activity. In recent years, MOFs have also been extensively employed to construct biosensors due to their unique merits.[^17b^, ^76a^, ^85^] In particular, the porous structure and high specific surface area of MOFs provide high catalytic activity. In addition, the designed shape and size as well as specific chemical interactions between guest molecules and MOFs endow them with high selectivity for their target molecules. Incorporating porphyrins into MOFs can remit the self‐quenching of porphyrins and combine the multiple functionalities of MOFs and porphyrins for biosensing with high sensitivity, resolution, and precision. To date, porphyrin‐based MOFs have been utilized for biosensing applications including electrochemical (EC), fluorescence, photoelectrochemical (PEC), electrochemiluminescence (ECL), and colorimetric biosensors. Various studies have demonstrated the possibility of the porphyrin‐based MOF‐based biosensors for detecting different biological substances and states, mainly including biomacromolecules, small molecules, ions, and pH values. Some typical examples of porphyrin‐based MOFs for biosensing are summarized in Tables 1–3.

#### 3.4.1 Biosensing of Biomacromolecules

**Figure 12:**
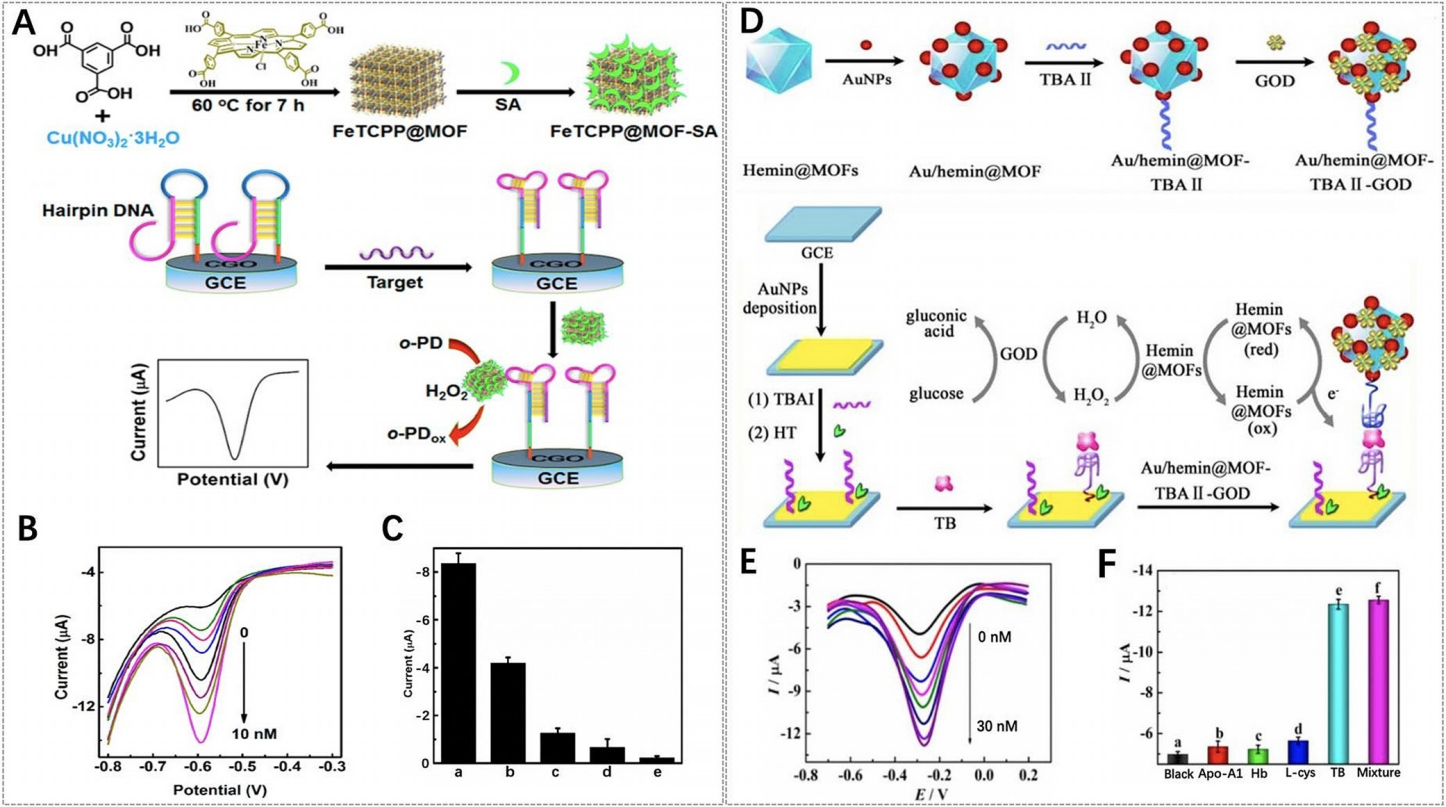
A) Synthesis of TCPP(Fe)@MOF‐SA and its electrochemical DNA sensing. B) Differential pulse voltammogram (DPV) responses of TCPP(Fe)@MOF‐SA to different concentrations of the target DNA from 0 to 10 nm. C) DPV responses of FeTCPP@MOF‐SA to 100 pm of different DNA sequences: a) target DNA, b) single‐base‐mismatched DNA, c) two‐base‐mismatched DNA, d) three‐base‐mismatched DNA, and e) random DNA in the catalytic system of *o*‐PD/H_2_O_2_. (A–C) Reproduced with permission.^[86a]^ Copyright 2015, American Chemical Society. D) Preparation of the hemin‐encapsulated Fe‐MIL‐88 based electrochemical biosensor and the TB detection principle. E) DPV of TB detection at different concentrations from 0 to 30 nm. F) Specificity of the biosensor toward different samples: a) no analyte, b) 100 nm Apo‐A1, c) 100 nm Hb, d) 100 nm l‐Cys, e) 10 nm TB, and f) 100 nm Apo‐A1+100 nm Hb+100 nm l‐cys+10 nm TB. (D–F) Reproduced with permission.^[26c]^ Copyright 2015, Royal Society of Chemistry.

DNA is the most essential genetic material of living beings, and various strategies have been proposed to detect DNA for diagnosis of diseases and biological processes. Among these strategies, EC biosensing has been extensively adopted for the detection of specific DNA owing to its high sensitivity and selectivity.^[85]^ For DNA electrochemical biosensors, porphyrin‐based MOFs with excellent electrocatalytic activity can be utilized as external probes for the production and amplification of the electrochemical signal.[^26d^, ^86^] For example, due to the peroxidase‐like catalytic activity of TCPP(Fe) metalloporphyrins, Lei et al. encapsulated TCPP(Fe) into HKUST‐1 and subsequently conjugated it with streptavidin (SA) to construct an smart signal‐transduction platform for hairpin DNA detection (Figure 12 A–C).^[86a]^ Here, a label‐free electrochemical DNA biosensor was designed by using TCPP(Fe)@MOF‐SA as a signal probe and hairpin DNA as an allosteric switch. The allosteric switch of hairpin DNA on the glassy carbon electrode (GCE) surface could form SA aptamer that could selectively bind the synthetic TCPP(Fe)@MOF‐SA via biorecognition affinity. Thus, the conjugated TCPP(Fe)@MOF‐SA on the GCE surface acted as a mimetic catalyst; in the presence of H_2_O_2_, it could greatly enhance the oxidation of *o*‐phenylenediamine (*o*‐PD) to 2,2′‐diaminoazobenzene, which could serve as a good electrochemical signal readout indicator. Such a “signal‐on” electrochemical biosensor was proved to detect hairpin DNA with a detection limit down to 0.48 fm (S/N=3), with a linear range over six orders of magnitude and good feasibility in complex serum matrixes.

On the other hand, porphyrinic MOFs for fluorescence biosensing have also been developed for DNA detection by utilizing the fluorescence quenching mechanism.[^33c^, ^87^] For example, Wang et al. used ultrathin Cu‐TCPP MOF nanosheets to design a fluorescence biosensor for rapid, sensitive, and multiplex detection of DNAs.^[87]^ Due to the large surface area and large number of accessible active sites on the nanosheets, three dye‐labeled ssDNA probes could be easily absorbed on the surface, leading to fluorescence quenching of the dye‐labeled ssDNA by Förster resonance energy transfer (FRET). When a specific target was hybridized with the ssDNA probes to form dsDNA, the formed dsDNA detached from Cu‐TCPP MOF nanosheets owing to the weak physical adsorption between Cu‐TCPP MOF nanosheets and dsDNA, and thereby the fluorescence “turned on”. Consequently, Cu‐TCPP MOF nanosheets exhibited excellent performance for quantitatively detecting pathogenic DNA with a detection limit in the pm range.

Immunoassays are of great interest for the clinical early diagnosis of various tumors, such as prostate specific antigen (PSA) analysis for diagnosis of prostate cancer. PEC bioanalysis is a potential bioanalytical method based on the photoinduced electron transfer (PET) processes among analyte, photoactive species, and electrode upon photoirradiation, which has been widely used in the detection of various biological molecules. Recently, Zhang et al. reported a new non‐enzyme PEC immunoassay using DNA‐mediated nanoscale PCN‐222 to detect PSA.^[88]^ The ss‐DNA‐tagged antibody (Ab‐DNA) was conjugated on PCN‐222 to form Ab‐DNA‐functionalized NMOF complexes (Ab‐DNA‐NMOFs). Ab‐DNA‐NMOFs, as an active photocathode PEC substrate, displayed a remarkably enhanced photocurrent response in the presence of dopamine (DA) in O_2_‐containing aqueous media. Further functionalized with polyamidoamine dendrimer, Ab‐DNA‐NMOFs could serve as an efficient PEC signal transduction system for non‐enzyme PSA immunoassays with hypersensitivity, a broad calibration range of 1 pg mL^−1^ to 10 ng mL^−1^, and a detection limit of 0.2 pg mL^−1^.

In addition, ECL, electrochemical redox‐induced light emission, possesses several advantages including low background interference, simple operation, and high sensitivity and space–time controllability. Han et al. proposed to use porphyrin@MOFs for PSA sensing via the ECL strategy.^[89]^ In this study, Fe‐MIL‐88 MOFs were used as a platform to load hemin and gold nanoparticles (Au NPs) and further graft G‐quadruplex DNAzyme for synergetic catalytic amplification of luminol. The pDNA‐Au‐Hemin‐MIL‐DNAzyme probes acted as quenchers and enhancers in the fabrication of a target‐induced ratiometric ECL biosensor for PSA detection. The results demonstrated that the pDNA‐Au‐Hemin‐MIL‐DNAzyme‐based biosensor was highly sensitive and could be used for the accurate analysis of PSA with a linear detection range from 0.5 to 500 ng mL^−1^ and a detection limit of 0.058 ng mL^−1^ (S/N=3).

Enzymes and proteins play important roles in metabolic regulation, cell growth, and other biological processes. Some of these biomolecules have been accepted as important biomarkers for early‐stage disease diagnosis, prognosis judgement, and pathogenesis studies. Several different types of porphyrin‐based MOFs have been used in the construction of electrochemical biosensors to enhance the electrochemical signal for enzyme or protein biosensing. Yuan et al. reported multifunctional hemin‐encapsulated Fe‐MIL‐88 MOFs, which were the first example for the construction of an electrochemical aptasensor for thrombin (TB) detection (Figure 12 D–F).^[26c]^ Such porphyrin@MOFs served as the support for the immobilization of recognition molecules (thrombin binding aptamer (TBA)) and were also conjugated with GOx for signal amplification. GOx oxidizes glucose into gluconic acid accompanied by the production of H_2_O_2_, which can be further electrocatalyzed by hemin to amplify the electrochemical signal. Such an electrochemical biosensor showed a relatively low detection limit of 0.068 pm and a wide linear range from 0.0001 nm to 30 nm with a high correlation coefficient of 0.9961, indicating that the constructed biosensor could be used to detect TB quantitatively. Lei et al. designed an electrochemical biosensor that was composed of iron metalloporphyrinic NMOFs (PorMOFs) for telomerase activity detection.^[90]^ PorMOFs exhibited excellent electrocatalytic activity towards O_2_ reduction, which was used to detect telomerase with a detection limit of 30 cells mL^−1^ and assess the telomerase activity even in single Hela cell. Moreover, porphyrin‐based MOFs were also applied to construct PEC and ECL biosensors for protein detection. For example, Zhang et al. developed a simple PCN‐222 based PEC biosensor for label‐free detection of a phosphoprotein (α‐casein).^[91]^ Shan et al. utilized zinc metalloporphyrinic MOFs (MOF‐525(Zn)) to develop an ECL biosensor for a highly sensitive protein kinase A (PKA) activity assay.^[92]^

#### 3.4.2 Biosensing of Small Molecules

Various porphyrin‐based MOF biosensors with peroxidase‐like activity have been developed for the detection of H_2_O_2_ and glucose, which are important biological indicators in clinical medicine. For instance, Liu et al. synthesized MIL‐101(Al)‐NH_2_ MOFs to anchor hemin for the formation of H@M composites, which showed peroxidase‐like activity for catalyzing oxidation of the peroxidase substrate 3,3,5,5‐tetramethylbenzidine (TMB) by H_2_O_2_ to produce a blue color in solution.^[93]^ Thus, H@M composites offered a simple, sensitive, and selective colorimetric approach for H_2_O_2_ determination, and the results showed that H_2_O_2_ could be measured at concentrations as low as 2 μm and the linear range was from 5 μm to 200 μm with a correlation coefficient of 0.997. Furthermore, by introducing GOx as a catalyst into H@M composites for the oxidation of glucose, a MOF‐based colorimetric biosensor was constructed for quantitative analysis of glucose in aqueous solution. The glucose biosensing was observed to have a linear range from 10 μm to 300 μm with a correlation coefficient of 0.996.

**Figure 13:**
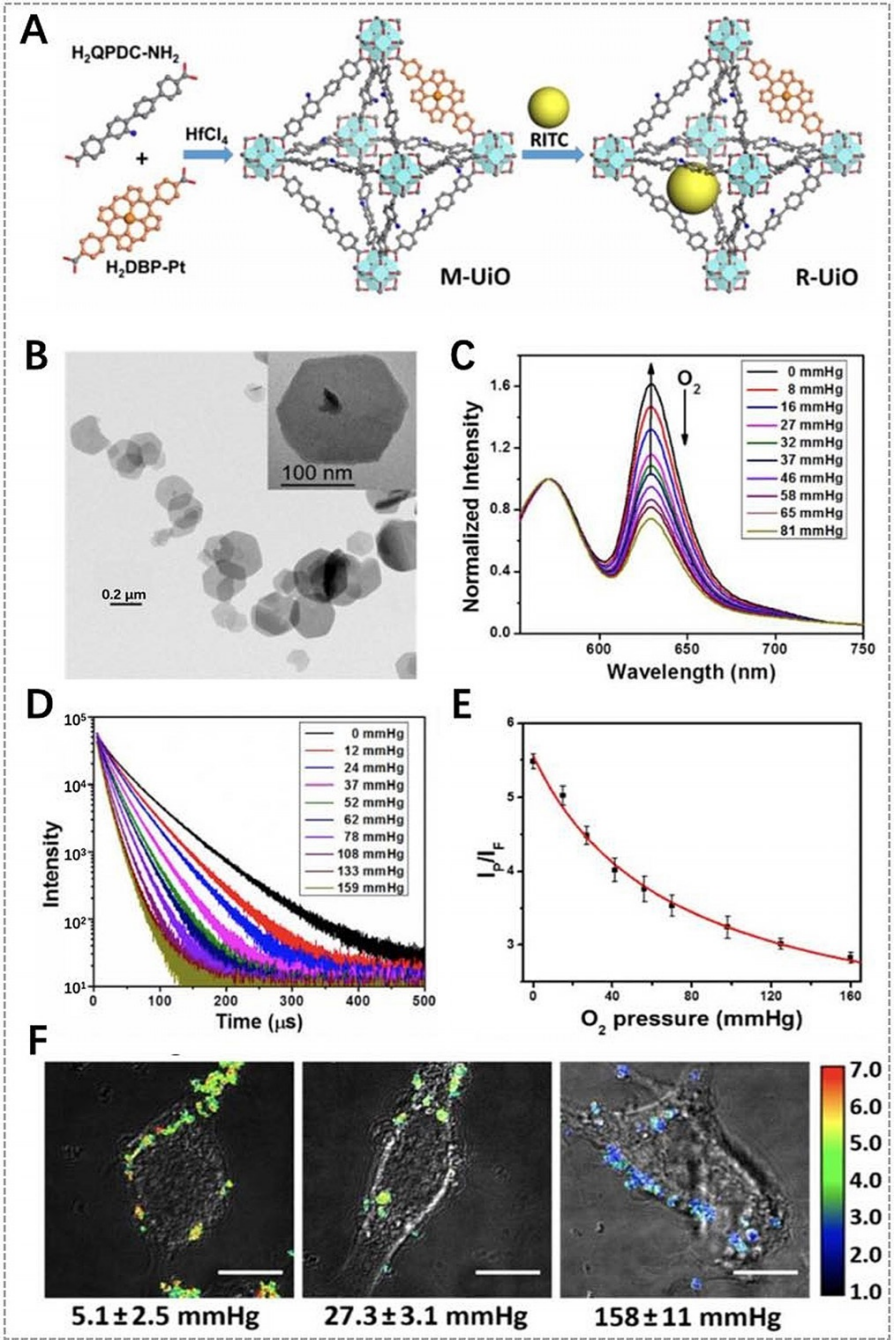
A) Synthesis of mixed‐ligand R‐UiO. B) TEM image of R‐UiO. C) Emission spectra and D) phosphorescent decay of R‐UiO in Hank's Balanced Salt Solution (HBSS) buffer under different oxygen partial pressures with excitation wavelengths of 514 nm and 405 nm. E) Calibration curve of the phosphorescence/fluorescence intensity of R‐UiO on confocal laser scanning microscopy (CLSM) under different O_2_ pressures. F) Ratiometric luminescence images of CT26 cells after treatment with R‐UiO under hypoxia, normoxia, and aerated conditions (from left to right) with an excitation wavelength of 514 nm. (A–F) Reproduced with permission.^[36b]^ Copyright 2016, American Chemical Society.

Monitoring and quantifying some gas levels (e.g., O_2_, H_2_S) in living cells is of great importance in clinical medicine, because the gas concentrations are correlated with many human physiological and pathological processes. For example, Lin et al. developed novel phosphorescence/fluorescence dual‐emissive NMOFs (R‐UiO) for ratiometric intracellular O_2_ quantification (Figure 13);^[36b]^ this was the first example of a MOF‐based intracellular O_2_ biosensor. R‐UiO NMOFs were synthesized by the coordination of the Pt^II^‐porphyrin linker DBP(Pt) as an O_2_‐sensitive probe and amino‐functionalized quaterphenyldicarboxylate linkers (QPDC‐NH_2_) with Hf ions. Then Rhodamine B isothiocyanate (RBITC) was covalently attached as an O_2_‐insensitive reference probe via thiourea bonds. DBP(Pt) and RBITC shared the same excitation energy, but the phosphorescence/fluorescence emissions did not interfere with each other, which allowed for the determination of intracellular O_2_ levels. When mouse colon carcinoma CT26 cells were treated with R‐UiO NMOFs under different O_2_ pressure (*p*O_2_) conditions at 4 mmHg (hypoxia), 32 mmHg (normoxia), and 160 mmHg (aerated condition), the analytic data from ratiometric luminescence images were 5.1±2.5 mmHg, 27.3±3.1 mmHg, and 158±11 mmHg, respectively. The results matched well with the preset *p*O_2_, suggesting the wide range and high accuracy of R‐UiO NMOFs as intracellular oxygen biosensors. Tang et al. reported aluminum–copper mixed‐metal porphyrinic MOFs (PAC) assembled from Al^3+^ nodes and TCPP(Cu) linkers as a heterogeneous fluorescence probe for the sensing of H_2_S in living cells.^[94]^ The Cu^2+^ centers in MOF frameworks induced the fluorescence quenching of TCPP porphyrins, which could be used as the H_2_S‐responding site for fluorescence “turn‐on” sensing of H_2_S. Consequently, this biocompatible fluorescence probe exhibited high selectivity and sensibility as well as low toxicity in in situ sensing of H_2_S in a physiological environment.

As a biochemical messenger, dopamine (DA) plays a critical role in neurotransmitters and neuromodulators in organisms that affect human activity. For measuring DA release from living cells, Yamauchi et al. designed an electrochemical biosensor using composite porphyrinic MOFs (MOF‐525‐PEDOT NTs),^[95]^ which was made up of conductive poly(3,4‐ethylenedioxythiophene) nanotubes (PEDOT NTs) coated with MOF‐525 nanocrystals. The synergistic conductivity and catalytic performance of MOF‐525‐PEDOT NTs enhanced the detection capability for DA with a linear concentration range (2 μm–270 μm) and detection limit (0.04 μm).

The rapid and sensitive detection of organophosphorus pesticides has gained more and more attention due to the environmental pollution and human health risks. Su et al. synthesized Cu‐H MOFs/NECF composites consisting of ball‐flower‐like Cu‐hemin MOFs grown on the fibers of 3D nitrogen‐containing melamine carbon foam (NECF) and immobilized AChE on Cu‐H MOFs/NEC for trichlorfon detection.^[96]^ The Cu‐H MOFs/NECF‐based biosensor exhibited great performance for trichlorfon detection with a broad linear range (0.25–20 ng mL^−1^), low detection limit (0.082 ng mL^−1^), and good stability, and thereby facilitated trace detection of organophosphorus pesticides.

#### 3.4.3 Ion Sensing

Several metal ions are essential elements in the human body and play vital roles in cellular activities, while their excess would be harmful for health. In particular, heavy metal ions are easily accumulated in the human body via the food chain, which thereby results in adverse effects. Obviously, it is of great importance to selectively detect metal ions in cells or in the environment. For porphyrin‐based MOFs, the coordination capability of metal ions in the porphyrin rings endows them with the ability to construct fluorescence “turn‐off” or “turn‐on” biosensors. In 2013, Li et al. proposed to use porphyrinic MOF‐525 as a fluorescence “turn‐off” biosensor for Cu^2+^ sensing.^[97]^ The porphyrin moieties in MOF‐525 provided accessible recognition sites for fluorescence quenching through energy/charge transfer to determine the amount of Cu^2+^ ions. MOF‐525 fluorescent probes showed great potential for rapid and specific detection of Cu^2+^ in practical water samples and for the study of copper‐related diseases like Wilson's and Alzheimer's diseases. Recently, a novel fluorescent porphyrinic MOF probe, where the UiO‐66(OH)_2_ MOF was encapsulated into PCN‐224, was developed by Lu et al. for detecting Cu^2+^ in complex water samples.^[98]^ Similarly, due to the fluorescence quenching ability of Cu^2+^ toward PCN‐224, this fluorescent probe exhibited excellent Cu^2+^ detection with a limit‐of‐detection (LOD) value as low as 0.068 nm, which is lower than the Cu^2+^ concentration limit in drinking water regulated by the WHO.

Compared to fluorescence “turn‐off” biosensors, fluorescence “turn‐on” biosensors can avert the wrong response to sensing signals and more conveniently identify in a dark background.^[36c]^ For example, Jiang et al. developed a fluorescence “turn‐on” biosensor based on Pd^II^ metalloporphyrinic MOFs (PCN‐222(Pd)) for Cu^2+^ sensing.^[99]^ Here, PCN‐222(Pd) was used as a catalytic Heck reaction system to produce highly fluorescent “turn‐on” signal in the presence of Cu^2+^, due to the stronger binding affinity of Cu^2+^ over Pd^2+^ to the nitrogen atoms in the porphyrin and the conversion of non‐fluorescent aniline to fluorescent ring‐closing product via Pd^0^‐catalyzed Heck reaction. Consequently, the PCN‐222(Pd) biosensor exhibited highly selective and sensitive sensing of Cu^2+^ in aqueous solution with a very low detection limit. In addition, porphyrinic MOFs were also utilized in the construction of electrochemical biosensors for detecting metal ions.^[100]^

Additionally, although anion sensing is more challenging, selective sensing of anions by porphyrin‐based MOFs has also been reported. For instance, Cao et al. developed Zr‐based MOFs with the mixed linkers 1,3,6,8‐tetra(4‐carboxylphenyl)pyrene (TBAPy) and TCPP (Zr(TBAPy)_5_(TCPP)) as a fluorescence “turn‐on” biosensor for S^2−^ sensing in aqueous environments.^[36c]^ The results showed that the fluorescence signal of Zr(TBAPy)_5_(TCPP) was intensified remarkably after addition of S^2−^, but other anions including SO_4_^2−^, CNS^−^, COOH^−^, Br^−^, I^−^, IO_3_^−^, F^−^, HSO_3_^−^, Cl^−^, and NO_3_^−^ did not alter its fluorescent intensity. Eddaoudi et al. encapsulated TMPyP(Pt) porphyrins in *rho*‐ZMOF for the selective sensing of various anions in aqueous and methanolic solutions.^[8c]^

#### 3.4.4 pH Sensing

Generally, pH plays an important role in maintaining the stability, normal morphology, and function of cells and organisms. Due to the spectral characteristics that change upon protonation/deprotonation of the pyrrole ring in porphyrins with a change in pH,^[101]^ porphyrin‐based MOFs could be used to construct pH sensors, showing great potential in pH monitoring, disease diagnosis, and even evaluating therapeutic efficacy. In 2013, Zhou et al. reported for the first time Zr‐based porphyrinic MOF PCN‐225 and metalloporphyrinic MOF PCN‐225(Zn) for pH sensing.^[35b]^ The results demonstrated that the fluorescence intensity of PCN‐225 has a strong correlation with the pH of the solution in the range of pH 0–10.2. The most acidic solution showed the weakest fluorescence, while the pH 10.2 solution gave the highest intensity. Furthermore, pH 7–10 was the most sensitive pH range for intensity response. Lu et al. developed an effective dual‐emission fluorescent MOF composite probe (RB‐PCN) that was constructed by the encapsulation of Fe_3_O_4_ nanoparticles in PCN‐224 followed by modification with RBITC.^[102]^ The results showed that RB‐PCN could sensitively detect a broad range of pH changes. It could also be used to monitor the pH changes in living cells by fluorescence confocal imaging and determine the pH values in actual water samples by fluorescence spectroscopy.

### 3.5 Other Applications of Porphyrin‐Based MOFs

In addition to the biomedical applications mentioned above, porphyrin‐based MOFs also have valuable applications in other biologically related fields. For example, Guo et al. synthesized spherical Cu‐TCPP(Cu) MOFs, and subsequently encapsulated Ag nanoparticles in MOFs to form Ag/Cu‐TCPP(Cu) composites for antibacterial application.^[103]^ The results showed that antibacterial effect of Ag/Cu‐TCPP(Cu) composites was superior to that of penicillin, and the cytotoxicity of Ag/Cu‐TCPP(Cu) composites was lower than that of naked Ag nanoparticles and Ag ions in vitro. Furthermore, Ag/Cu‐TCPP(Cu) composites could also significantly promote wound healing in vivo.

More recently, Qu et al. utilized ultrathin Cu‐TCPP(Fe) MOF nanosheets to physically adsorb GOx to construct a composite nanocatalyst for in vivo wound healing.^[104]^ Here, Cu‐TCPP(Fe)/GOx could continuously convert nontoxic glucose into gluconic acid and H_2_O_2_ through the action of GOx, and the subsequent pH decrease from 7 to 3–4 could dramatically activate the peroxidase‐like activity of Cu‐TCPP(Fe) MOF nanosheets. Subsequently, the produced H_2_O_2_ could be further transformed to toxic ^.^OH radicals by the Fenton reactions of Cu and Fe ions. In vitro and in vivo experiments showed that this cascade catalytic conversion of nontoxic glucose to toxic ^.^OH radicals endowed the Cu‐TCPP(Fe)/GOx nanocatalyst with robust antibacterial activity and negligible biotoxicity.

## 4 Toxicity and Safety Concerns

Regarding porphyrin‐based MOFs for biomedical applications, their potential toxicity is of high concern. Considering the construction of MOFs from metal nodes and organic linkers, it is a priority to choose nontoxic or hypotoxic building components to improve the biocompatibility and reduce the toxicity. Fortunately, porphyrins as biocompatible motifs are beneficial for the MOFs’ biosafety. However, the toxicity of MOFs was found to be closely related to their metal nodes.^[76a]^ The biocompatible metal ions (e.g., Zr, Fe, Zn, Ca, Mg) are possible nodes for the construction of biofriendly porphyrin‐based MOFs due to their acceptable toxicity for biomedical applications.^[106]^ Especially for the synthesis of composite porphyrinic MOFs, the additional functional components also need to be considered for potential damage to the tissues and organisms. In addition to the composition of porphyrin‐based MOFs, the physicochemical natures of MOFs including size distribution, shape, surface chemistry, and dose‐dependent properties are also important factors in terms of toxicology.[^106^, ^107^] All these parameters strongly affect the absorption, distribution, metabolism and excretion, biodegradation, and clearance of porphyrin‐based MOFs, and further determine their potential toxicity in vivo. To date, these aspects of porphyrin‐based MOFs have not been fully investigated, while the multifunctionality has been deeply explored in most biomedical studies.

Understanding the intricate interactions between the physicochemical properties, biological performance, and biosafety could contribute to the successful translation to practical biomedical applications. Therefore, the relevant studies of porphyrin‐based MOFs in biomedical applications need to be developed in depth. Firstly, it is important to use various progressive characterization techniques to accurately determine the physicochemical parameters of MOFs (e.g., size, shape, surface chemistry, porosity) and reasonably evaluate their merits and limitations in order to understand and ultimately modulate the structure–function relationship for MOFs.^[108]^ Next, investigating the correlation between MOFs and biological systems is the basis for correlating the MOFs’ features with the toxicity results. Recently, Faria et al. proposed the MIRIBEL (Minimum Information Reporting in Bio‐Nano Experimental Literature) standard for different studies on bio–nano interactions, which normalizes material characterization, biological characterization, and experimental details for enhancing reproducibility and quantitative comparisons of bio–nano materials.^[109]^ In the development of porphyrin‐based MOFs for biomedical applications, such standard should be referenced to facilitate safety evaluation and material optimizations. Furthermore, great progress has been made with respect to mechanism‐based hazard assessment of nanomaterials and advanced tools have emerged for their safety evaluation.^[110]^ Therefore, not only for porphyrin‐based MOFs, researchers need to accelerate the use of emerging methods and tools, combining research results with bioinformatics, to scientifically and quantitatively correlate the data outcome of biomaterials and their toxicity results for their practical application in biomedicine.

## 5 Conclusions and Perspectives

Porphyrins and their derivatives have been often used as photosensitizers for PDT, fluorophores for fluorescence imaging, and sensors for biosensing due to their excellent photophysical properties. As a new type of porous materials, MOFs possess high porosity, tunable structures, multifunctionality, biocompatibility, and biodegradability. Studies have demonstrated that the construction of porphyrin‐based MOFs can not only overcome the limitations of free porphyrins like instability and self‐quenching issues in physiological environments, but also integrate the functionalities of MOFs and porphyrins as well as improve the physicochemical properties. In this Review, recent achievements and progress in the construction of porphyrin‐based MOFs and their biomedical applications have been highlighted and discussed.

To date, various methods have been used to construct porphyrin@MOFs, porphyrinic MOFs, and composite porphyrinic MOFs in order to meet the requirements of biomedical applications. Among them, many strategies including heavy‐atom substitution of porphyrinic MOFs, controlling the particle size, controllable design, and modification with targeting moieties or functional components have been proposed to enhance the therapeutic efficacy of porphyrin‐based MOFs in PDT. Additionally, the rational design of functional components in MOFs or adjuvant agents makes it possible to interfere with survival mechanisms and alter the tumor microenvironment to achieve better results in terms of PDT efficacy. Obviously, the optimized construction of porphyrin‐based MOFs shows great potential for enhanced PDT of tumors.

Another efficient strategy for enhancing therapeutic efficacy is the implementation of multimodality therapy in a single system to overcome the limitations of single‐modality therapy. Porphyrin‐based MOFs have exhibited a great capacity for combining PDT with other therapy modalities, including chemotherapy, PTT, immunotherapy, and gas therapy, to achieve synergistic effects for maximizing therapeutic efficacy and improving safety. However, the majority of current studies focus on dual‐modality therapies, but there are few reports on tri‐ or more modality therapy based on porphyrin‐based MOFs, which might offer more significant therapeutic effects.

Porphyrin‐based MOFs have also been developed for imaging‐guided therapy to further optimize the therapeutic process and thereby enhance therapeutic efficacy. Due to the excellent fluorescent properties and photosensitization of porphyrins, porphyrin‐based MOFs could realize fluorescence imaging‐guided therapy. On the other hand, metal nodes in MOFs or the incorporation of contract agents in MOFs endow porphyrin‐based MOFs with the potential for MRI‐, CT‐, PAI‐ or PTI‐guided therapy. Such “all‐in‐one” nanoplatforms provide more accurate diagnosis and guidance for treatment, and are of great attractive for tumor therapy.

Besides the exploration of tumor therapy functions, porphyrin‐based MOFs have been utilized to construct biosensors for detecting various biological species, such as biomacromolecules, small molecules, and ions as well as pH values and gases. Owing to the combined excellent physicochemical properties of MOFs and porphyrins, porphyrin‐based MOFs types of biosensors show significant performance in biosensing with high sensitivity and selectivity, acceptable reproducibility, good stability, and biocompatibility. Some porphyrins including hemin, TCPP, and its metal derivatives are often used to construct porphyrin‐based MOF type biosensors. However, it can be anticipated that the biosensing performance could be further improved with more interesting porphyrins. On the other hand, porphyrin‐based MOF type biosensors for cell sensing and in vivo sensing need further investigation for the development of biosensors in living biological systems.

Despite the growing number of impressive progress reports on porphyrin‐based MOFs, their development in biomedical applications is still at the early stage and faces many challenges. For porphyrin@MOFs, the porphyrin loading capacity and premature leakage and self‐quenching of porphyrins should be considered during the synthesis process. Actually, porphyrinic MOFs are more promising candidates for biomedical applications. However, the influence of size, shape, composition, and surface chemistry of porphyrin‐based MOFs on therapeutic efficacy and biosensing ability should be further optimized to meet the requirements of personalized therapy, diagnosis, and detection. On the other hand, the design of “all‐in‐one” nanoplatforms based on composite porphyrinic MOFs would be an efficient strategy to enhance therapeutic efficacy in tumor therapy and biosensing ability. Actually, researchers should establish straightforward synthesis protocols for these designed MOF materials, as the simpler the preparation, the more likely it is that the product will be relevant for biomedical applications in the real world. Finally, it should be re‐emphasized that thoroughly and deeply investigating the correlation between the fundamental parameters of porphyrin‐based MOFs and their biosafety including the pharmacokinetics, biodistribution, biocompatibility, long‐term toxicity, and clearance from the body, is crucial for possible clinical translations. In conclusion, porphyrin‐based MOFs have great potential for applications in tumor therapy and biosensing, and more advances and progress in the biomedical field will be achieved in the future.

## Conflict of interest

The authors declare no conflict of interest.

## Biographical Information

*Jiajie Chen is currently studying Materials Science and Engineering at University of Shanghai for Science and Technology and has been in the Master's program since 2017. His research interests focus on the synthesis of multifunctional porphyrinic MOFs for biomedical applications in cancer therapy and biosensing*.

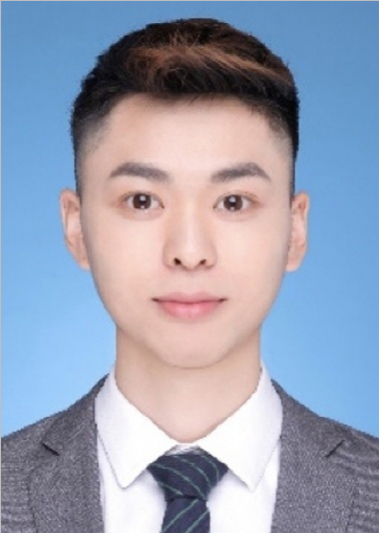

## Biographical Information

*Yufang Zhu studied Materials Physics and Chemistry and received his PhD degree at Shanghai Institute of Ceramics*, *Chinese Academy of Sciences in 2006. He worked as an Alexander von Humboldt research fellow at Technical University Dresden (Germany) and an ICYS postdoctoral fellow at National Institute for Materials Science (Japan) from 2006 to 2010. Since 2011 he has been Professor of Materials Science and Engineering at University of Shanghai for Science and Technology, and Professor at Shanghai Institute of Ceramics, Chinese Academy of Sciences since 2019*.

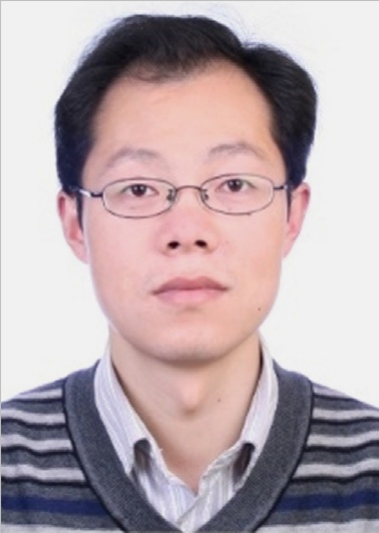

## Biographical Information

*Stefan Kaskel studied Chemistry and received his PhD degree in Tübingen in 1997. After a postdoctoral stay as a Feodor Lynen fellow of the Alexander von Humboldt foundation in the group of J. D. Corbett, he obtained his habilitation degree in 2003 at Bochum University on the design and functionality of new porous materials. From 2002 to 2004 he was also a group leader at the Max Planck Institute for Coal Research. Since 2004 he has been Professor of Inorganic Chemistry at Dresden University of Technology and since 2008 he has also headed the Department of Chemical Surface Technology at the Fraunhofer Institute for Material and Beam Technology (IWS), Dresden*.